# Determinants of measles persistence in Beijing, China: A modelling study

**DOI:** 10.1017/S0950268823001322

**Published:** 2023-08-22

**Authors:** Jianjiu Chen, Wenyi Zhang, Yong Wang, Wan Yang

**Affiliations:** 1 Department of Epidemiology, Mailman School of Public Health, Columbia University, New York, NY, USA; 2 Chinese PLA Center for Disease Control and Prevention, Beijing, China; 3 Herbert Irving Comprehensive Cancer Center, Columbia University Medical Center, New York, NY, USA

**Keywords:** measles (rubeola), mathematical modelling, particle filtering, catch-up vaccination, migration

## Abstract

In Beijing, the capital of China, routine measles mass vaccination has been in place for decades with high coverage; and since the 2000s, catch-up vaccination programmes have been implemented for migrant workers coming to the city. However, measles epidemics in Beijing persisted. Here, we explored the contributing factors of persistent measles transmission in Beijing using an epidemic model in conjunction with a particle filter. Model inputs included data on birth, death, migration, and vaccination. We formulated a series of hypotheses covering the impact of migrant influx, early waning of maternal immunity, and increased mixing among infants; we compared the plausibility of the hypotheses based on model fit to age-grouped, weekly measles incidence data from January 2005 to December 2014, and out-of-fit prediction during 2015–2019. Our best models showed close agreement with the data, and the out-of-fit prediction generally captured the trend of measles incidence from 2015 to 2019. We found that large influx of migrants with considerably higher susceptibility likely contributed to the persistent measles transmission in Beijing. Our findings suggest that stronger catch-up vaccination programmes for migrants may help eliminate measles transmission in Beijing.

## Introduction

Measles is a highly infectious and severe viral infection (estimated case fatality ratio ~ 2% in low- and middle-income countries [1]). Thanks to mass vaccination, measles incidence has decreased substantially worldwide in recent years, making global eradication plausible [2]. However, to date, none of the six World Health Organization regions have achieved and maintained measles elimination [3]. Moreover, the COVID-19 pandemic has disrupted measles vaccination programmes, further creating an environment for measles persistence [4].

China has made considerable efforts to control and eliminate measles [5, 6]. The national Expanded Program on Immunization began in 1978, and for more than a decade, has reported vaccination coverage for measles exceeding 95% – the critical level for elimination [5]. From 2004 to 2010, in addition to the two-dose routine vaccination programme, the government conducted supplementary immunization activities (SIAs), administering ~289 million doses to children aged between 8 months and 14 years [5, 6]. These efforts substantially reduced measles incidence but did not eliminate the disease in China [5].

Beijing is the capital of China. Routine measles vaccination programme in Beijing started in the 1970s; since the 2000s, the city has also implemented various catch-up vaccination campaigns targeting migrants, a subpopulation growing rapidly after 1995 [7]. Despite these efforts, measles epidemics persisted in Beijing [7]. In a previous study, we applied an epidemic model in conjunction with a particle filter to yearly measles incidence data to infer the long-term measles transmission dynamics from 1951 to 2004 in three locations including Beijing [8]. Here, we modified this model-filter system to study measles transmission dynamics in Beijing from 2005 to 2014 using weekly incidence data. To examine factors driving persistent measles transmission in Beijing, we formulated a range of hypotheses covering the potential impact of migrant influx, early waning of maternal immunity, and increased mixing among infants, based on emerging evidence [9, 10]. We assessed these hypotheses and underlying mechanisms based on model fit to data during 2005–2014, and out-of-fit prediction for data during 2015–2019.

## Methods

### Data

**Figure 1.**
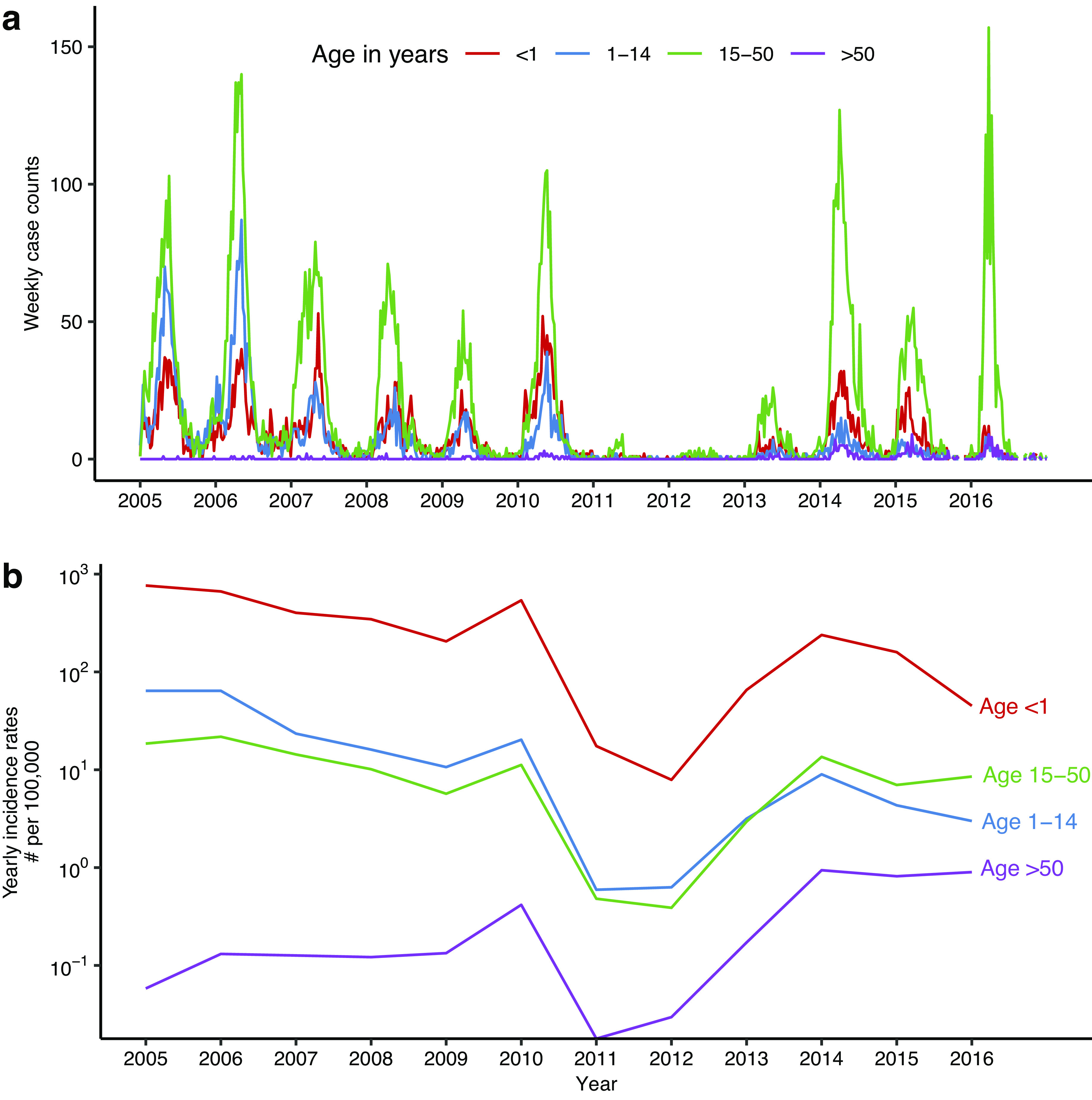
Measles incidence in Beijing during 2005-2016. Incidence data by week (A) and by year (B) for four age groups.

Weekly measles incidence in 4 age groups (i.e., <1, 1–14, 15–50, and > 50 years) from 2005 to 2016 (627 weeks; Figure 1) came from the China Information System for Disease Control and Prevention (CISDCP) [11], a web-based real-time disease reporting system collecting patient-case reports for all notifiable diseases, including measles, from all medical institutions in China since 2004. Yearly incidence rates for all ages combined during 1995–2004 (used to generate prior estimates) came from Li et al. [7], and that during 2017–2019 came from the CISDCP. Data sources on birth, death, migration, and vaccination are detailed in Sections 1 and 2 of the Supplementary Materials.

### Model-filter system

#### Epidemic model

The epidemic model (Supplementary Figure S1) followed the age-structured susceptible-exposed-infectious-removed (SEIR) construct [8, 12], per Equation (1)):(1)

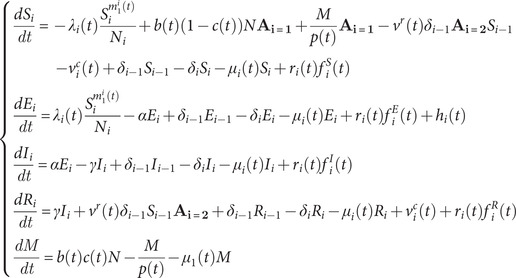

 where 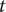 is time in days. 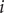 ( 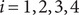 ) represents 4 age groups: <1, 1–14, 15–50, and > 50 years. For Group 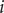 , 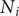 is the population size, which is divided into those who are susceptible, 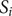 ; exposed (latently infected but not yet infectious), 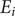 ; infectious, 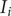 ; or recovered or immunised, 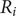 . *M* represents infants with maternal immunity. 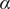 is the rate of progression from latent infection to infectiousness and 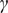 is the recovery rate. 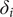 and 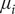 are the aging and natural death rates ( 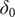 and 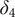 were set to 0).

The force of infection, 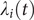 , is given by(2)

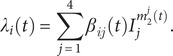

 where 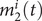 is the mixing ratio of infectious individuals in Group *i*; 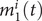 is the corresponding mixing ratio of susceptible individuals. These exponents represent the extent of inhomogeneous mixing, with the value 1 representing homogeneous mixing. The exponents are further scaled by 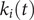 to test hypotheses related to differential mixing among migrants and infants (see Sections 4.4 and 4.6 of the Supplementary Materials), per(3)

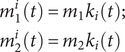



The transmission rate, 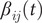 , accounts for seasonality and school term-time mixing per(4)

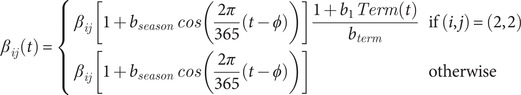

 where 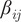 is the mean transmission rate from Group 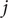 to Group 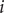 ; 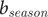 is the amplitude of sinusoidal forcing, and 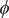 represents the day of a year when the sinusoidal forcing reaches the maximum. As in previous work [13], for school-age children (here Group 2), we included a school term-time forcing 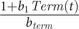 to model the impact of congregation during school terms. 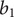 is the amplitude of the school term forcing, and 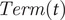 is given by(5)

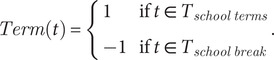



Based on the school schedule in China, 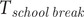 includes a summer break that lasts for 7 weeks and ends on August 31st, and a winter break that lasts for 5 weeks and ends on the 14th day after the Lunar New Year (LNY). 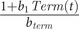 averages 1 in a calendar year.

The epidemic model includes 4 age groups and thus needs 16 (=4 × 4) parameters for all 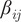 ’s. To reduce the number of parameters, the 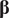 matrix is formulated using 6 parameters:(6)

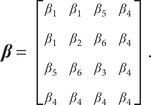



Here, we assumed the transmission rate among infants (aged <1) equals that between infants and older children (aged 1–14), that is, 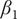 . We also assumed all transmission rates associated with Group 4 are the same (i.e., 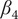 ) because the oldest age group contributes less to measles dynamics and also has less social contact.

The basic reproductive number, 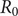 , in this age-structured model is related to the 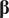 matrix per the next generation matrix method [13]:(7)

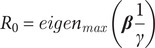

 where 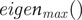 is the function that gives the largest eigenvalue of the matrix. We reparametrised the model based on Equation (7) and included 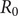 for estimation by setting 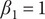 and estimating the relative magnitude of 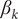 ( 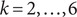 ).

Relating to births and maternal immunity, 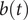 is the birth rate per data, and 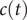 is the proportion of infants with maternal immunity (set to 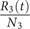 , i.e., the immunity level of the age group of new mothers). 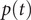 is the mean duration of maternal immunity, and varied by hypothesis (Section 4.5 of the Supplementary Materials). 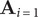 and 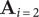 are indicator functions that were set to 1 for 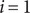 and 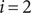 , respectively; otherwise, these functions were set to 0. In Line 1 of Equation (1)), for infants (Group 1), 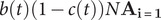 represents newborns without maternal immunity; 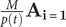 represents infants who are initially protected by maternal immunity gradually becoming susceptible as this protection wanes.

For vaccination, we included both routine and catch-up vaccination. For simplicity, the routine vaccination in the model only includes 1 dose at age 1; this roughly captures the impact of the 2-dose measles vaccination programme, in which the first dose is administered at 8 months and the second at 18–24 months of age [6]. To account for vaccine effectiveness, we computed the immunization rate, 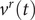 , combining vaccination rates and effectiveness for both doses. In Lines 1 and 4 of Equation (1)), for 1- to 14-year-olds (Group 2), 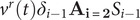 represents the number of children turning age 1 (i.e., 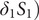 and getting immunised. 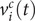 represents the number of individuals acquiring immunity from catch-up vaccination (see Supplementary Materials for details).

We also accounted for migration. Specifically, 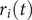 represents the net number of migrants (i.e., the change in population size excluding births and deaths, due to a lack of detailed data) in Group 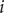 ; 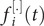 represents the fraction of migrants to Group *i* with a given disease status as specified by the superscript. The setting for these migration-related terms varied by hypothesis (Sections 4.1 and 4.3 of the Supplementary Materials). Lastly, 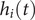 models migration-related case importation (see specific settings in Section 4.2 of the Supplementary Materials).

#### Model inference (parameter estimation)

To estimate the model state variables ( 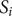 , 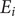 , 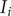 , 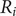 , 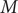 ) and parameters ( 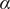 , 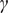 , 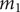 , 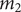 , 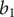 , 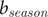 , 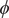 , 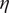 , 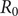 , and 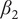 to 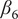 ), we ran the epidemic model in conjunction with a modified particle filter [14]. Briefly, we initialised the model-filter system at the start of 1995, that is, before the large influx of migrants. The initial model state and parameter values (i.e., prior distributions) for the particles (i.e., model replica) came from our previous work [8] and the literature [15] (see Supplementary Materials, Section 3). The system then estimated the model state sequentially from 1995 to 2014 via prediction-update cycles. In the prediction stage, the system integrates the particles forward stochastically with a daily time-step according to the epidemic model (Equation (1)); this generates the prediction, or the prior, for the next time step. In the update stage, the system computes the likelihood for each particle, and combines it with the prior to compute the posterior per Bayes’ rule.

To compute the likelihood, we modelled the reported incidence in Group 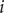 during reporting period 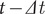 to 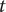 ( 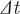 is 1 year during 1995–2004 and 1 week during 2005–2014 based on data availability), *Y_i,t_*, using a Gaussian distribution:(8)

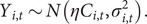

 where 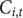 is the corresponding model-estimated measles cases (including unreported cases) and 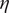 is the reporting rate (estimated by the filter). Following our previous work for Beijing [8], we assumed the reporting rate increased linearly before 2005 to reflect the improvement in disease surveillance. We set the annual increase to 0.0037, corresponding to a 20% increase from 1951 to 2004. 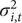 is the observational error variance. During 1995–2004, only yearly incidence combining all ages (Y_t_) was available. Accordingly, we aggregated model estimated cases across all ages and, given the large year-to-year variation, simply scaled the observational error variance 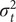 to the reported incidence for the same year in addition to an arbitrary baseline of 100, per(9)

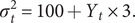



For 2005–2014, weekly incidence data were available for the 4 age groups. We used these data for inference and computed the observational error variance as(10)

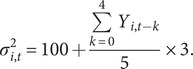



As a sensitivity analysis, we tested smaller observational errors, that is, replacing the baseline of 100 with 50 and scaling factor of 3 with 1.

During filtering, we applied space reprobing, a technique to explore the state space to a larger extent to prevent the particles from being trapped in a sub-state space (a challenge known as particle impoverishment) [16]. We reprobed the parameter space during 1995–2004 when only yearly data were available, and during 2005 when the filter switched to using weekly data. In addition, the net migration estimates used in the model likely underestimated the influx of susceptible migrants, particularly during 2011–2014 when the estimates were low (Supplementary Figure S2); as such, we also reprobed 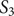 (i.e., susceptibles aged 15–50, the age group where most migrants were in) during the first four weeks after the LNY (i.e., the likely timing of worker migration) in 2011–2014.

### Models to test different hypotheses

Using the base epidemic model described above, we modified related model terms to test six sets of hypotheses (Table 1); each set, including several competing hypotheses, represents a potential mechanism underlying the measles epidemics in Beijing, as described below and detailed in the Supplementary Materials.

**Table 1.** Tested hypotheses

Hypothesized mechanism	Hypothesis label	Brief description
Higher migrant susceptibility	MigSusBase	Migrants as susceptible as the population of Beijing
	MigSusMedium	Migrants as susceptible as the population of Shandong
	MigSusHigh	Migrants 50% more susceptible than the population of Shandong
Case importation (i.e., seeding) due to migrant influx	BaseSeed	Seeding after the LNY fixed at a low level
	MediumSeed	Seeding after the LNY scales with migrant population size (lower intensity)
	StrongSeed	Seeding after the LNY scales with migrant population size (higher intensity)
Timing of migrant influx	MigConst	Constant migrant influx
	MigLNY4	Migrant influx ended 4 weeks after the LNY
	MigLNY12	Migrant influx ended 12 weeks after the LNY
Increased migrant mixing	MigMixBase	No periodic change in migrant mixing
	MigMixMedium	Migrant mixing increased from the LNY to the end of March (*k* = 1.025)^a^
	MigMixHigh	Migrant mixing increased from the LNY to the end of March (*k* = 1.05)^a^
Shorter duration of maternal immunity	Imm180	180 days of maternal immunity
	Imm150	150 days of maternal immunity
	Imm120	120 days of maternal immunity
Increased infant mixing	InfantMixBase	No increase in infant mixing
	InfantMixMedium	Increased infant mixing (*k* = 1.025)^a^
	InfantMixHigh	Increased infant mixing (*k* = 1.05)^a^

LNY, Lunar New Year.

a *k* represents the extent of increase in mixing intensity.

#### Rationale and description

##### (1) Higher migrant susceptibility

Given the disparities in vaccination programmes across China and the substantial migrant influx [7], it was possible that higher susceptibility levels of migrants than those in Beijing contributed to the epidemics. Therefore, we tested three hypotheses on migrant susceptibility: The migrants were (i) as susceptible as the population in Beijing (referred to as MigSusBase; Table 1); (ii) as susceptible as the population in Shandong, a proxy for migrants as Shandong is an important source of migrant workers and estimates were available from our previous work [8] (MigSusMedium); and (iii) 50% more susceptible than the population in Shandong (MigSusHigh).

##### (2) Case importation (i.e., seeding) due to migrant influx

Migrant workers typically left their hometowns to seek jobs in Beijing shortly after the LNY. This is roughly the period when annual measles epidemics occurred. The epidemics, therefore, might have been driven by importation of infected migrants (i.e., seeding). We tested three seeding scenarios by varying the specification of 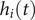 in Equation (1), that is, fixing the seeding at a low level (BaseSeed) and scaling the seeding with migrant influx (MediumSeed and StrongSeed).

##### (3) Timing of migrant influx

After the LNY when intense migration typically occurs, a sudden large influx of susceptible migrants could quickly increase the susceptible pool and exacerbate the impact. We thus tested the timing and intensity of migrant influx, including constant influx (MigConst, i.e., distributing the net migration, 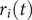 in Equation (1)), evenly over time) and migration concentrating within 4 or 12 weeks following the LNY (MigLNY4 and MigLNY12, i.e., distributing the net migration 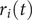 to only those weeks).

##### (4) Increased migrant mixing

During the first few months after LNY, there could be a large number of temporary job seekers (likely not captured by the net migration data used in our model), who typically shared close living space in the city, leading to a period with potentially increased mixing among migrants. As such, we tested the impact of increased migrant mixing shortly after the LNY. In the tested scenarios, we either increased the time-varying mixing parameters for those aged 15–50, that is, 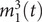 and 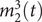 in Equation (3), from the LNY to the end of March (MigMixMedium and MigMixHigh) or assumed no change in these mixing parameters (MigMixBase).

##### (5) Shorter duration of maternal immunity

Recent serological studies have showed that maternal measles antibodies wane earlier in infants born to mothers who acquired immunity via vaccination than those via natural infections [9]. In addition, a substantial proportion of measles cases occurred among infants (<1 year; on average 22% during 2005–2016; Table 2). We thus hypothesised that, after decades of mass vaccination, the duration of maternal immunity may have been shortened; this in turn may have rendered more infants susceptible prior to receiving their first vaccine dose at 8 months of age and contributed to measles persistence. To test this, we set 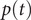 in Equation (1), the duration of maternal immunity, to 180, 150, or 120 days (Imm180, Imm150, and Imm120).

**Table 2.** Age distribution of measles cases by year and cases combing all ages in Beijing from 2005 to 2019

Age	2005	2006	2007	2008	2009	2010	2011	2012	2013	2014	2015	2016	2017	2018	2019
*% of cases by age groups*															
<1	21%	18%	22%	26%	26%	32%	29%	19%	22%	20%	25%	7%	–^a^	–	–
1–14	32%	30%	18%	16%	18%	16%	13%	17%	12%	8%	7%	5%	–	–	–
15–50	47%	52%	60%	57%	55%	51%	58%	61%	64%	69%	64%	82%	–	–	–
>50	0%	0%	0%	0%	1%	1%	0%	3%	2%	3%	4%	5%	–	–	–
*Number of cases*															
All ages	3404	3762	2273	1799	1109	2491	96	75	557	2384	1321	1250	64	103	55

a During 2017–2019, measles cases by age groups were not available.

##### (6) Increased infant mixing

Infants may cluster together in various venues such as hospitals and daycare centres, which may increase the risk of measles transmission among infants and caretakers. For instance, a literature review suggests that hospital-acquired measles infection is an important mode of transmission in the post-vaccine era [10]. A measles outbreak investigation in China also identified a hospital as an important venue of transmission [17]. Therefore, we also hypothesised that mixing intensity among infants is higher than other age groups. In the tested scenarios, we either increased the mixing parameters for those aged <1, that is, 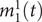 and 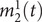 in Equation (3), (InfantMixMedium and InfantMixHigh) or assumed no increase in these mixing parameters (InfantMixBase).

#### Comparison procedure

**Figure 2.**
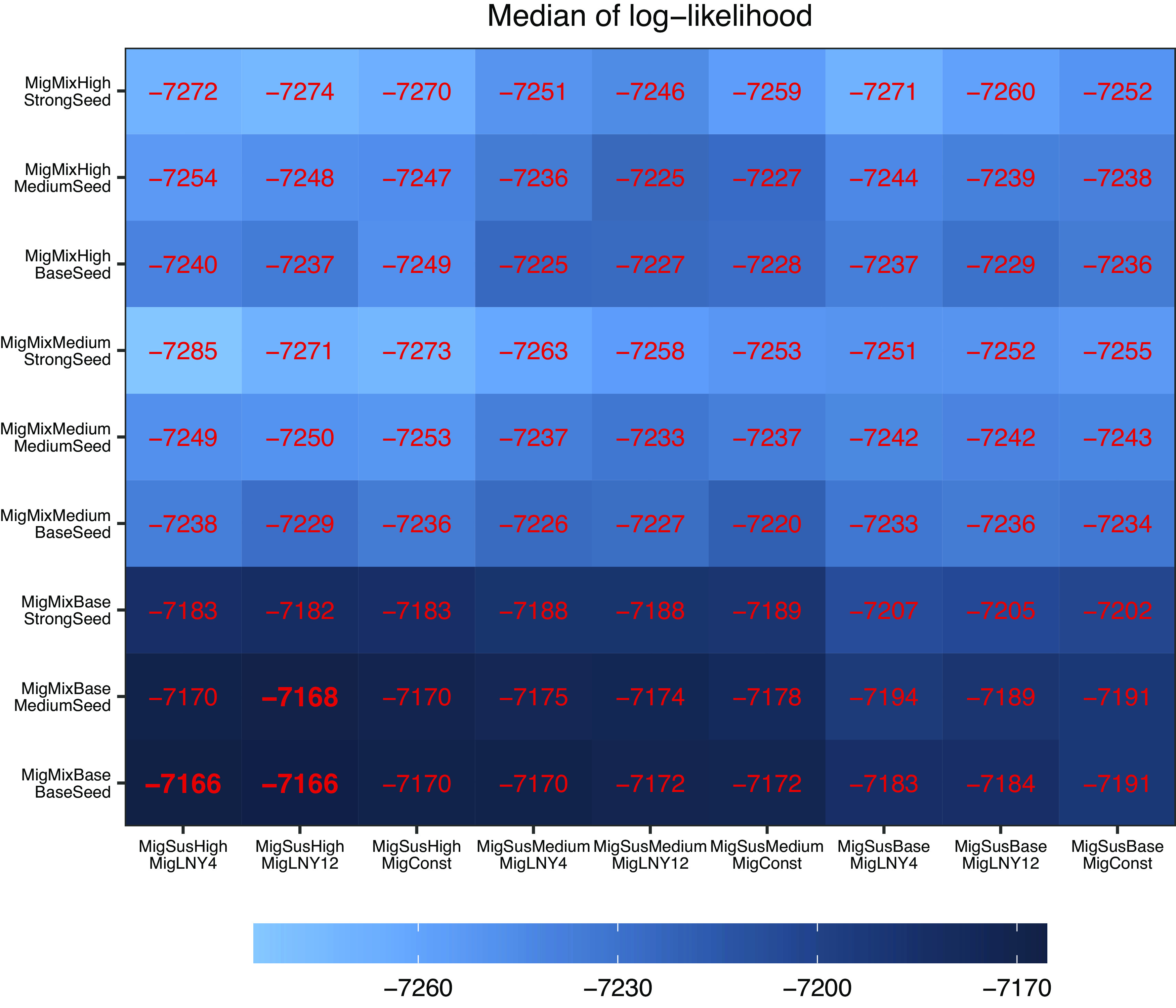
Comparing migrant-related hypotheses based on medians of log-likelihood. We formulated four sets of migrant-related hypotheses (increased migrant mixing [MigMixBase/MigMixMedium/MigMixHigh], seeding intensity [BaseSeed/MediumSeed/StrongSeed], higher migrant susceptibility [MigSusBase/MigSusMedium/MigSusHigh], and timing of migrant influx [MigConst/MigLNY12/MigLNY4]), and tested all combinations of the four sets (*n* = 81, i.e., the number of cells in the Fig). For each model (or hypothesis combination), we conducted 100 model inference runs using a model-filter system, and show the median of the 100 log-likelihood estimates in red. For infant-related hypotheses, all models tested here assumed Imm180 and InfantMixBase as baseline scenarios. See Table 1 for a summary of all hypotheses.

**Figure 3.**
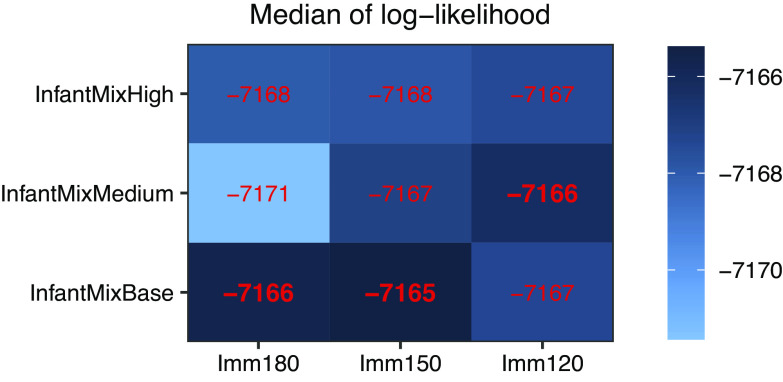
Comparing infant-related hypotheses based on medians of log-likelihood. We formulated two sets of infant-related hypotheses (increased infant mixing [InfantMixBase/InfantMixMedium/InfantMixHigh] and duration of maternal immunity [Imm180/Imm150/Imm120]), and tested all combinations of the two sets (*n* = 9, i.e., the number of cells in the Fig). For each model (or hypothesis combination), we conducted 100 model inference runs using a model-filter system, and show the median of the 100 log-likelihood estimates in red. For migrant-related hypotheses, all models tested here assumed MigMixBase, BaseSeed, MigLNY4, and MigSusHigh, because the previous comparison step identified this combination as one of the most plausible scenarios. See Table 1 for a summary of all hypotheses.

Given the large number of combinations across the six sets of hypotheses, we compared the aforementioned hypotheses in two steps that focussed on migrant-related and infant-related hypotheses, respectively. In Step 1, we tested all combinations of the four sets of migrant-related hypotheses (n = 81; Figure 2), while setting the two sets of infant-related hypotheses to the baseline scenarios, that is, Imm180 and InfantMixBase. In Step 2, setting the migrant-related hypotheses at the most plausible scenario identified from Step 1, we further tested all combinations of the two sets of infant-related hypotheses (n = 9; Figure 3).

### Model comparisons

We compared the different models based on their goodness-of-fit (GOF) during 2005–2014 (with weekly data for all four age groups). We considered two GOF measures: (1) log-likelihood and (2) root-mean-square-error (RMSE) for the four age groups. To account for model and filter stochasticity, we ran the inference process for each model (or combination of hypotheses) 100 times, each with 8000 particles. Due to the large variance of both GOF measures, we used boxplots to show their distributions for qualitive comparisons.

In instances where competing models fitted the data equally well, we simulated the models forward from the start of 2015 and compared their out-of-fit prediction. We initialised the model for simulation using the posteriors at the end of 2014. In addition, as the error in state variables grows during the course of simulation, we reset 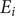 and 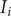 at the start of each year to the average of reported cases in Group 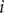 in the first two weeks of each year divided by the reporting rate, when such weekly data were available (i.e., for 2015 and 2016). In addition, as noted above, the net migration estimates likely failed to capture migration-induced susceptibility changes, particularly during 2015–2019 (see the near zero or negative net migration in Supplementary Figure S2). To address this, we used the model-filter system and weekly data during 2015–2016 to estimate the changes in susceptibility among those aged 15–50, the group including most migrants (i.e., 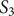 ); we reprobed 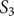 in broader state spaces to allow the posterior of 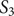 to reflect migration-induced changes. We then adjusted 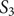 to match the posterior of 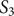 for the out-of-fit simulation during 2015–2016. We did not estimate or adjust 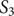 for 2017 onwards because of the lack of weekly data for those years.

## Results

### Characteristics of the measles epidemics in Beijing during 2005–2019

Despite the high vaccination coverage (Supplementary Figure S2), measles epidemics recurred in Beijing almost annually during the study period (Figure 1a). Epidemic intensity was substantially lower in 2011 and 2012 (likely due to the national SIA in September 2010), resurged during 2013–2016, and declined again after 2017 (Table 2). A seasonal pattern with an increase in cases in early spring was evident for years with a substantial outbreak (Figure 1a). Those aged 15–50 accounted for the majority of cases (the proportion of cases in this age group ranged from 47% to 82%; Table 2). This was in contrast with the pre-vaccine era, when measles infections predominantly occurred among young children [13]. In addition, infants (age < 1 year) saw large numbers of cases (7% to 32% of the total), and the highest age-specific incidence rates among all age groups (Figure 1b).

### Impact of migrants

We first tested four sets of migrant-related hypotheses in combination (i.e., higher migrant susceptibility, seeding intensity, timing of migrant influx, and increased migrant mixing). Compared with models assuming no increase in migrant mixing (MigMixBase), models assuming increased migrant mixing (MigMixMedium and MigMixHigh) had worse model fit (lower likelihood and higher RMSE; Figure 2 and Supplementary Figures S3–S5). Models assuming stronger seeding due to migration (MediumSeed and StrongSeed) also had worse model fit than those assuming baseline seeding (Figure 2 and Supplementary Figures S3–S5). As such, we focussed on models assuming no increase in migrant mixing or seeding intensity (see the bottom row in Figure 2 and Supplementary Figure S4 and the nine rows of boxplots at the bottom of Supplementary Figures S3 and S5). Among these models, increasing migrant susceptibility improved model fit in terms of log-likelihood and RMSE for those aged 1–14 and 15–50 (log-likelihood: MigSusBase < MigSusMedium < MigSusHigh). In comparison, model settings on migrant influx timing had a minor impact on model fit (Supplementary Figures S3 and S5) or out-of-fit predictions (Supplementary Figures S6 and S7), except for Year 2016 where the MigLNY4 model had smaller under-prediction than the other two settings (Supplementary Figure S6). Taken together, these findings suggest migrant susceptibility substantially affected the overall measles epidemic dynamics in Beijing and that migrants might have a considerably higher susceptibility level than the locals.

### Impact of Infant-related factors (maternal immunity and mixing intensity)

Models assuming shorter maternal immunity duration (represented by Imm180, Imm150, and Imm120) had smaller RMSE for those aged under 1 year (Supplementary Figure S8B). However, these competing hypotheses generated similar model performances when assessed by all other measures, that is, log-likelihood, RMSE for the other age groups, and out-of-fit prediction (Supplementary Figures S8–S10). In addition, models assuming different intensity of infant mixing also generated similar results on all measures of model performances (Supplementary Figures S8–S10).

### Models accounting for higher migrant susceptibility best captured measles epidemic dynamics in Beijing during 2005–2019

As detailed above, three settings substantially affected model performance, and those assuming higher susceptibility among migrants and baseline seeding and migrant mixing had the best model performance. Thus, we considered all related models (i.e., MigSusHigh, BaseSeed, and MigMixBase) best models. The sensitivity analysis, in which we used smaller observation errors, generated results (Supplementary Figure S11) that were similar with the main results.

**Figure 4.**
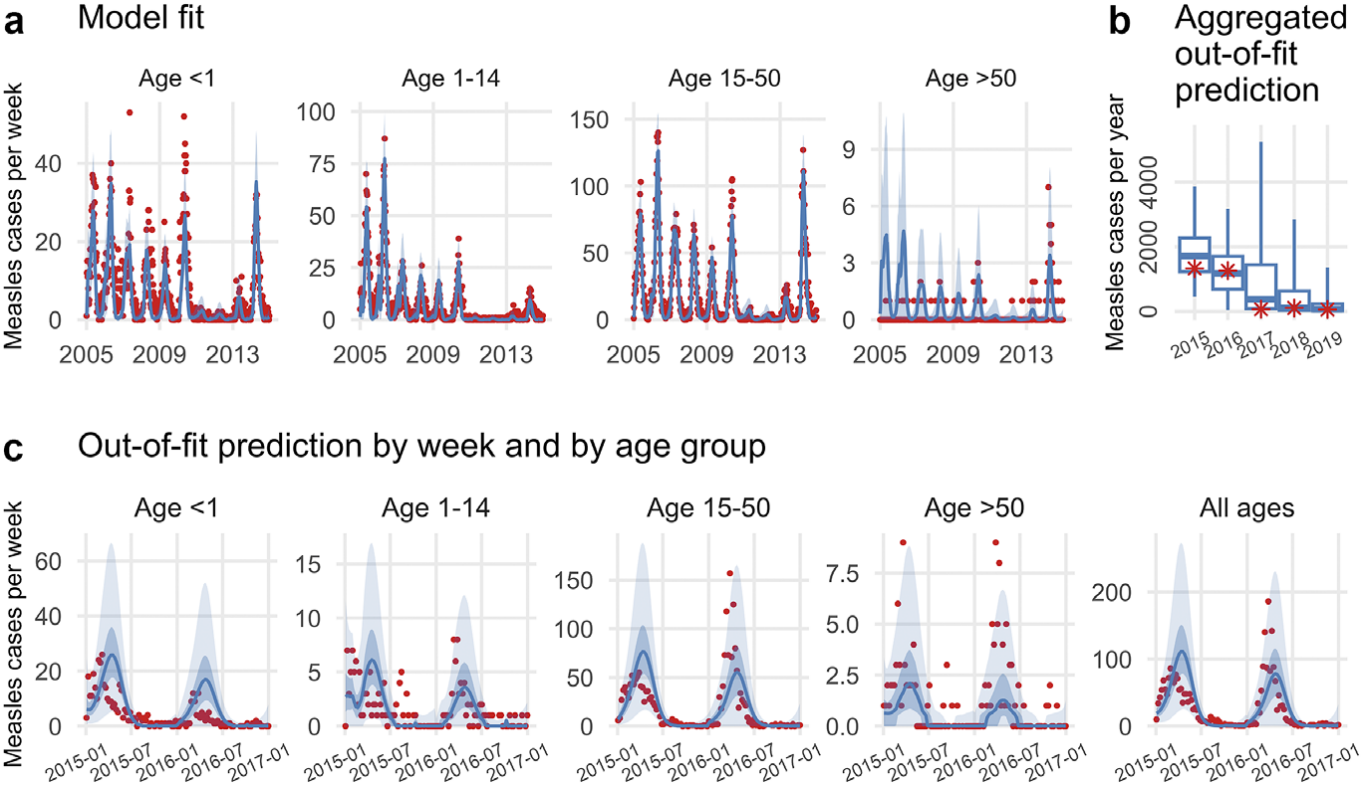
Model performance of one of the best models. This model assumed MigSusHigh, BaseSeed, MigLNY4, MigMixBase, Imm180, and InfantMixBase. See Table 1 for a summary of all hypotheses. The model fit is shown in (a); out-of-fit predictions are shown in (b) and (c). In (a), the red dots represent reported weekly case counts; the blue lines and surrounding areas represent the mean and 95% CrI of the posterior. In (b), the red asterisks represent reported yearly case counts; the whiskers, box edges, and thick horizontal segment in the middle represent the 2.5th (or 97.5th) percentile, interquartile range, and median of predicted cases, respectively. In (c), the red dots represent reported weekly case counts; blue lines show the median of predicted cases; surrounding darker blue areas indicate interquartile range, and lighter blue areas indicate 2.5 to 97.5 percentiles.

Figure 4 shows the model fit and out-of-fit prediction for one of these best models. The model was able to capture the annual epidemics and dynamics among all age groups during the 2005–2014 training period (Figure 4a). The parameter estimates (Supplementary Figure S12) were generally consistent with those reported in the literature [18–20]; for instance, estimated 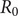 was 16.0 (50% credible interval [CrI]: 14.6–17.3; 95% CrI: 12.0–20.0), latent period ( 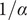 ) was 8.1 days (50% CrI: 7.8–8.4; 95% CrI: 7.3–9.0), and infectious period ( 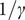 ) was 5.0 days (50% CrI: 4.7–5.3; 95% CrI: 4.2–5.8). In addition, the out-of-fit prediction, when aggregated across all ages to yearly totals, correctly captured the downward trend of measles incidence from 2015 to 2019 (Figure 4b). For years 2015–2016 when weekly data are available, the model generally captured the overall and age group-specific epidemic dynamics (Figure 4c).

## Discussion

To examine determinants of measles persistence in Beijing, we tested a series of hypothesised mechanisms including the impact of migrant influx, early waning of maternal immunity, and increased mixing among infants. Overall, model inference suggests the influx of migrants and their higher susceptibility likely contributed to the persistent measles transmission in Beijing. Models accounting for these key factors were able to recreate measles epidemic dynamics during 2005–2014, and predict epidemics during 2015–2019.

Foremost, model inference estimated higher susceptibility for migrants and supports vaccinating migrants to control measles. Indeed, catch-up vaccination campaigns for migrants have been implemented in Beijing since the 2000s [7, 21], which likely mitigated but did not prevent the outbreaks. Our model inference suggests two important gaps. First, while a large number of migrants were vaccinated via catch-up vaccination, many were likely missed and sufficient to sustain endemic transmission (e.g., on average ~ 378 thousands vaccinated each year during 2005–2010 [21] vs. ~737 thousands estimated net migration, not including those uncounted). As such, stronger catch-up vaccination programmes might be needed. Second, measles epidemics occurred shortly after the LNY (January–February), but catch-up vaccination campaigns were conducted during March–May [21]. Given the delay, catch-up vaccination in coordination with local public health agencies to vaccinate migrants in source regions before they leave for big cities like Beijing might be more effective.

Unexpectedly, migrant influx timing did not substantially affect model dynamics. The similar model performances may be due to the use of net migration estimates, which likely missed a large number of temporary migrants who failed to find a job to remain in the city. Future investigation using more detailed migration data is thus warranted to further examine the impact of the LNY intense migration. In addition, this data limitation may have led to overestimation of the gap in susceptibility levels between migrants and locals (estimated 28% susceptible for migrants aged 15–50 and 16% for their local counterpart in the best models). In reality, it is likely that a migrant influx of larger volume and lower susceptibility fuelled the outbreaks.

Four other hypothesised mechanisms – increased case importation due to migrant influx, increased migrant mixing, shorter duration of maternal immunity, and increased infant mixing – also did not substantially affect model dynamics. These findings suggest these potential mechanisms likely had a smaller impact on measles dynamics during our study period. For instance, in Beijing and China overall, the first measles vaccine is administered at 8 months of age [7], earlier than in many other places (e.g., at 12–15 months in the USA [22]); this earlier vaccine administration and the relatively low number of cases under 1 may explain the lack of a major impact from shortening maternal immunity and increasing infant mixing. Nonetheless, we cannot rule out these potential mechanisms, due to several study limitations as noted below. Further investigation on these mechanisms is thus warranted.

We recognise several limitations in our study. First, here measles incidence data combined cases from migrants and local residents. Thus, we were unable to test the migration-related hypotheses using migrant-specific data. Nevertheless, by modelling the long-term measles dynamics including a period before large migrant influx, we were still able to identify migration and the higher susceptibility of migrants as a key contributor to measles persistence. Second, the lack of migrant-specific data may have limited our ability to detect more specific impact of migration, for example, changes in migrant mixing intensity over time. Third, the incidence data were likely affected by under-reporting [5] and likely more so for migrants. Lastly, using population-level incidence data and models, we were also unable to capture some of the more nuanced system dynamics. In particular, to examine the potential impact of enhanced mixing and transmission among infants via venues like paediatric hospitals and daycares and subsequent transmission to caretakers, individual or household level data may be needed.

In summary, our findings have revealed key mechanisms of measles persistence in Beijing and suggested potential prevention strategies. Model inference found considerably higher susceptibility of migrants likely contributed to persistent measles transmission in Beijing, suggesting the need for stronger catch-up vaccination programmes for migrants.

## Supporting information

Chen et al. supplementary material Chen et al. supplementary material

Chen et al. supplementary material Chen et al. supplementary material

Chen et al. supplementary material Chen et al. supplementary material

## Data Availability

The measles incidence data are subject to restriction. To access these data and/or to seek permission for its use, please contact the Data-Center of China Public Health Science (http://www.phsciencedata. cn/Share/en/data.jsp?id = f17e302d-b0ef-4573-ae43-4fa55e7de9f5a&show = 0) or email data@chinacdc.cn.

## Supplementary material

The supplementary material for this article can be found at https://doi.org/10.1017/S0950268823001322.
