# Supplementary material for: Determinants of measles persistence in Beijing, China: A modelling study
Source: Epidemiol Infect. 2023 Aug 22;151:e144. doi: 10.1017/S0950268823001322 (PMC10540187; doi:10.1017/S0950268823001322)
Supplement: Chen et al. supplementary material [file S0950268823001322sup001.docx]

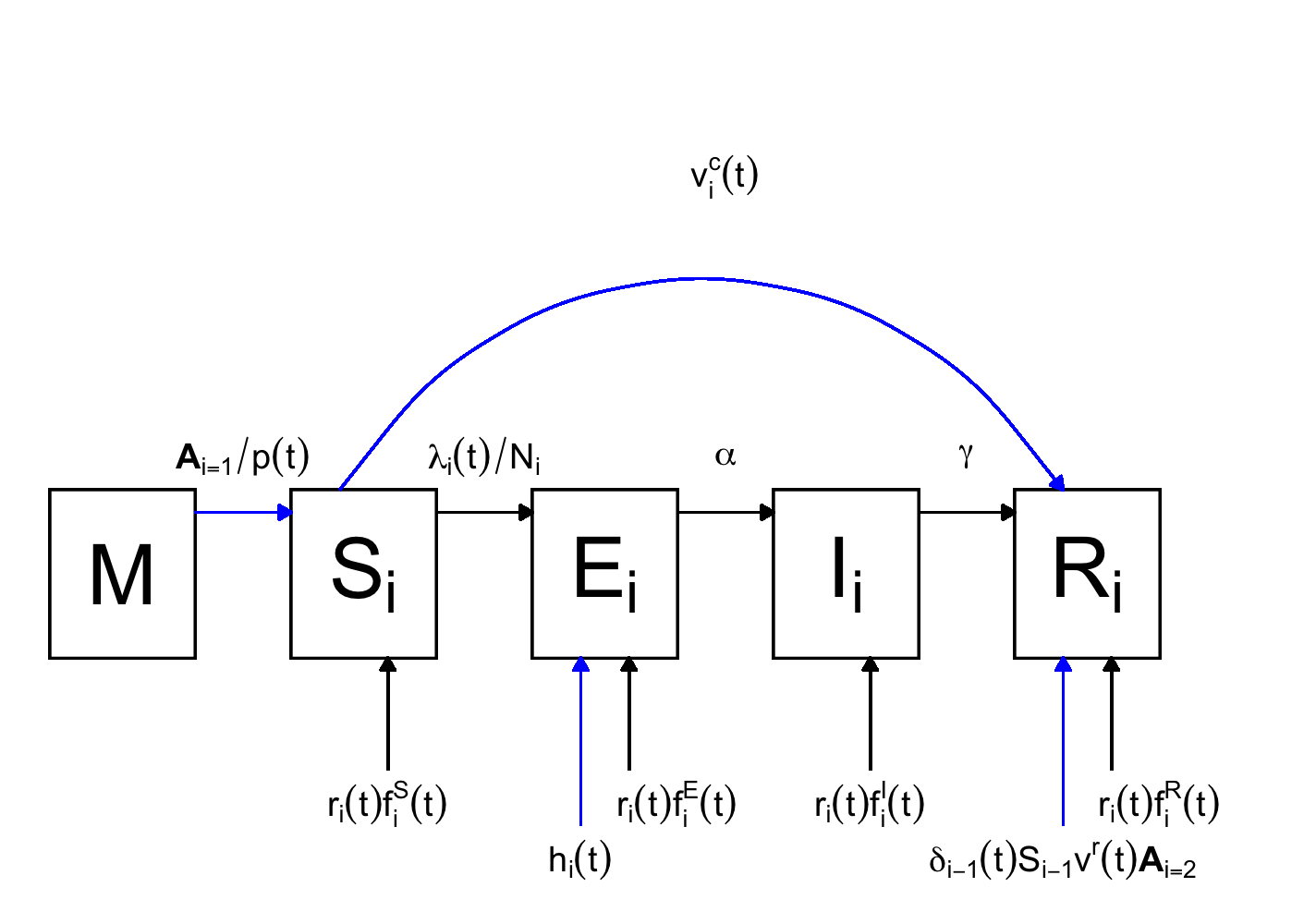


**Fig S1. Model schematic.** Modeled measles transmission follows the Susceptible-Exposed-Infectious-Removed (SEIR) dynamics. The model is age-structured, with age indicated by $i$. For simplicity, only one age group $i$ is shown; birth, aging, and death are modeled but not shown here. Compartment M represents newborns with maternal immunity. $\lambda_{i}(t)$ is the force of infection; $N_{i}$, the population size in age $i$. $\alpha$ is the rate of progression from the exposed to the infectious; $\gamma$, the recovery rate. $r_{i}(t)$ is the rate of migration; $f_{i}^{[.]}(t)$ is the fraction of migrants in compartment $[.]$ ($[.]\in\{S,E,I,R\}$). $p(t)$ is the mean duration of maternal immunity. $\mathbf{A}_{\mathbf{i=k}}$ is an indicator function that equals 1 when $i=k$ and 0 otherwise. $v_{i}^{c}(t)$ is the number of people vaccinated during catch-up or supplementary vaccination programs. $\delta_{i-1}(t)S_{i-1}v^{r}(t)\mathbf{A}_{\mathbf{i=2}}$ represents routine vaccination (note both routine vaccination doses are administered around 1 year of age in Beijing, i.e., when individuals from group 1 are aging into group 2; $\delta_{i-1}(t)$ and $v^{r}(t)$ are the aging rate and routine vaccine coverage, respectively). $h_{i}(t)$ represents seeding due to migration, and applies only if $i\in\{1,2,3\}$. Black arrows apply to all age groups; blue arrows apply to a subset of age groups.


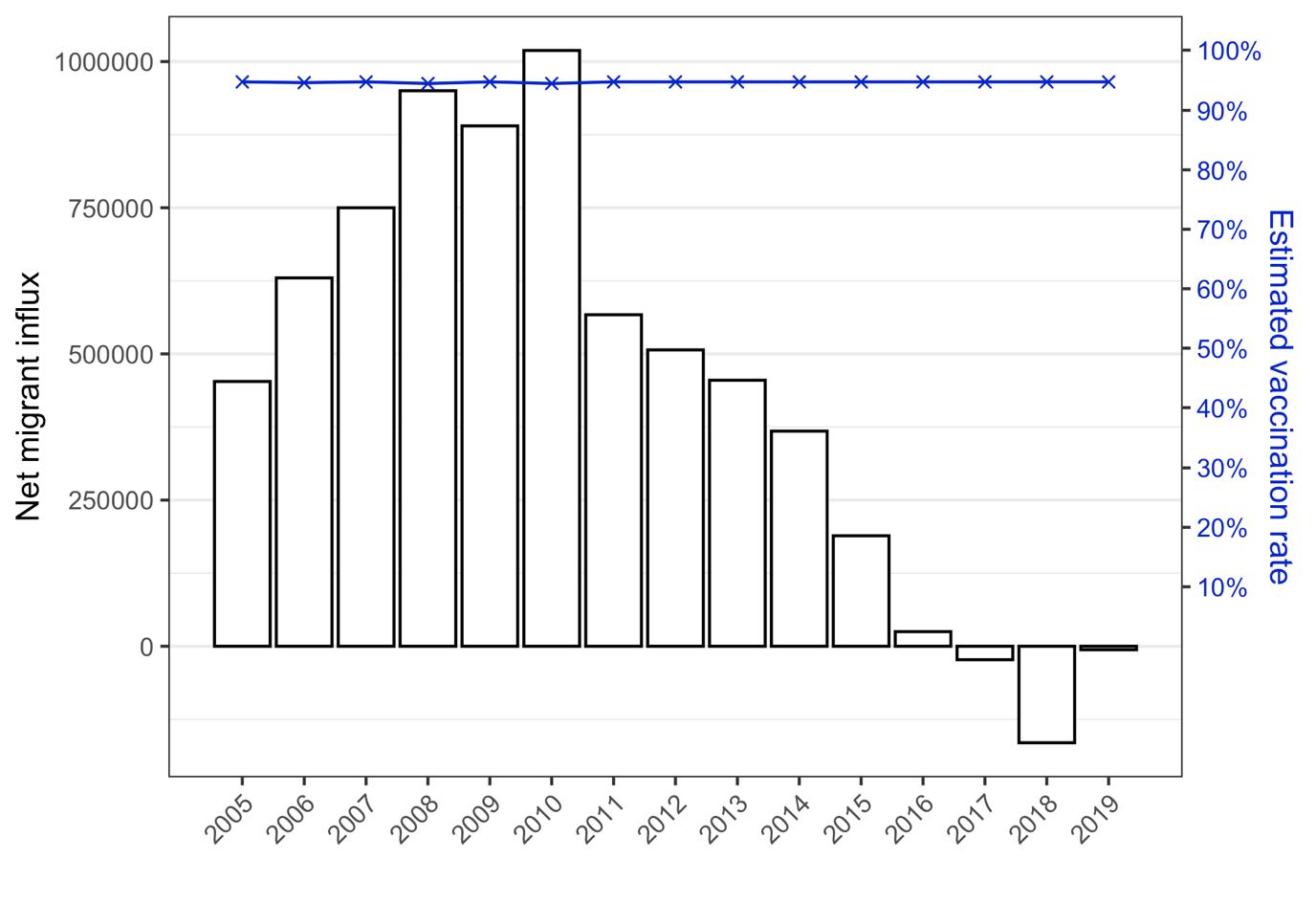


**Fig S2. Trends of net migration and vaccination rate in Beijing during 2005-2019.** Due to a lack of detailed migration data, the models used net migration (histograms) as a proxy of total migration. This likely underestimated migrant influx, and more so for years after 2011 when net influx decreased to low or negative values. We estimated vaccination rate (blue line) by combining coverage of 2-dose measles vaccine and vaccine efficacy (90% and 95% for 1- and 2-doses, respectively; see details in Section 2.1 of Supplementary Materials).


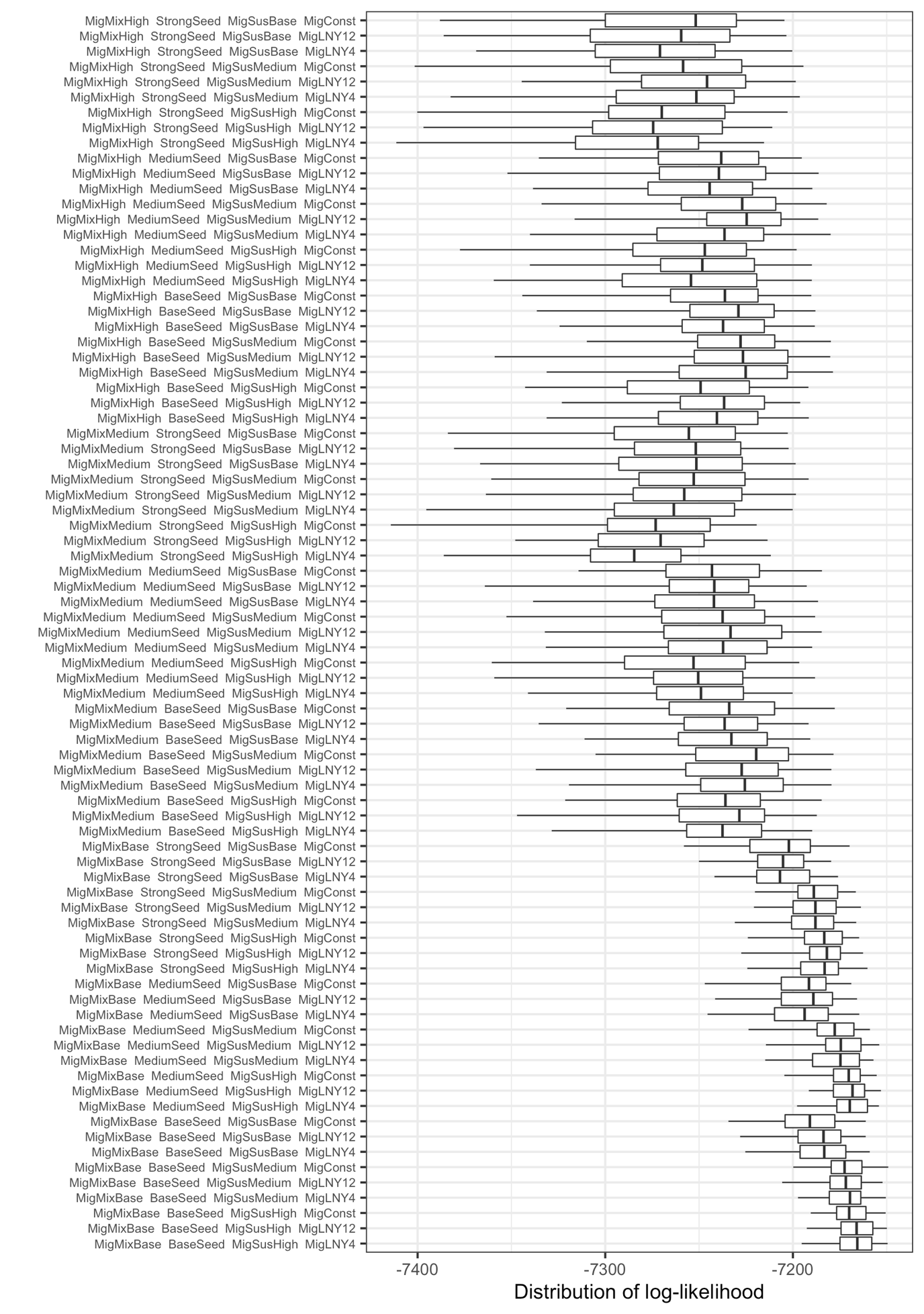


**Fig S3. Comparing migrant-related hypotheses based on distributions of log-likelihood.** We formulated four sets of migrant-related hypotheses (increased migrant mixing, seeding intensity, higher migrant susceptibility, and timing of migrant influx; corresponding to the 1st, 2nd, 3rd, and 4th terms in y-axis), and tested all combinations of the four sets (n=81, i.e., the number of boxplots). For each model (or hypothesis combination), we conducted 100 model inference runs using a model-filter system, and show the distribution of log-likelihood using boxplots (whiskers = 95% interval, box edges = Interquartile range, and thick middle bar = median). For infant-related hypotheses, all models tested here assumed Imm180 and InfantMixBase as baseline scenarios. See Table 1 for a summary of all hypotheses.


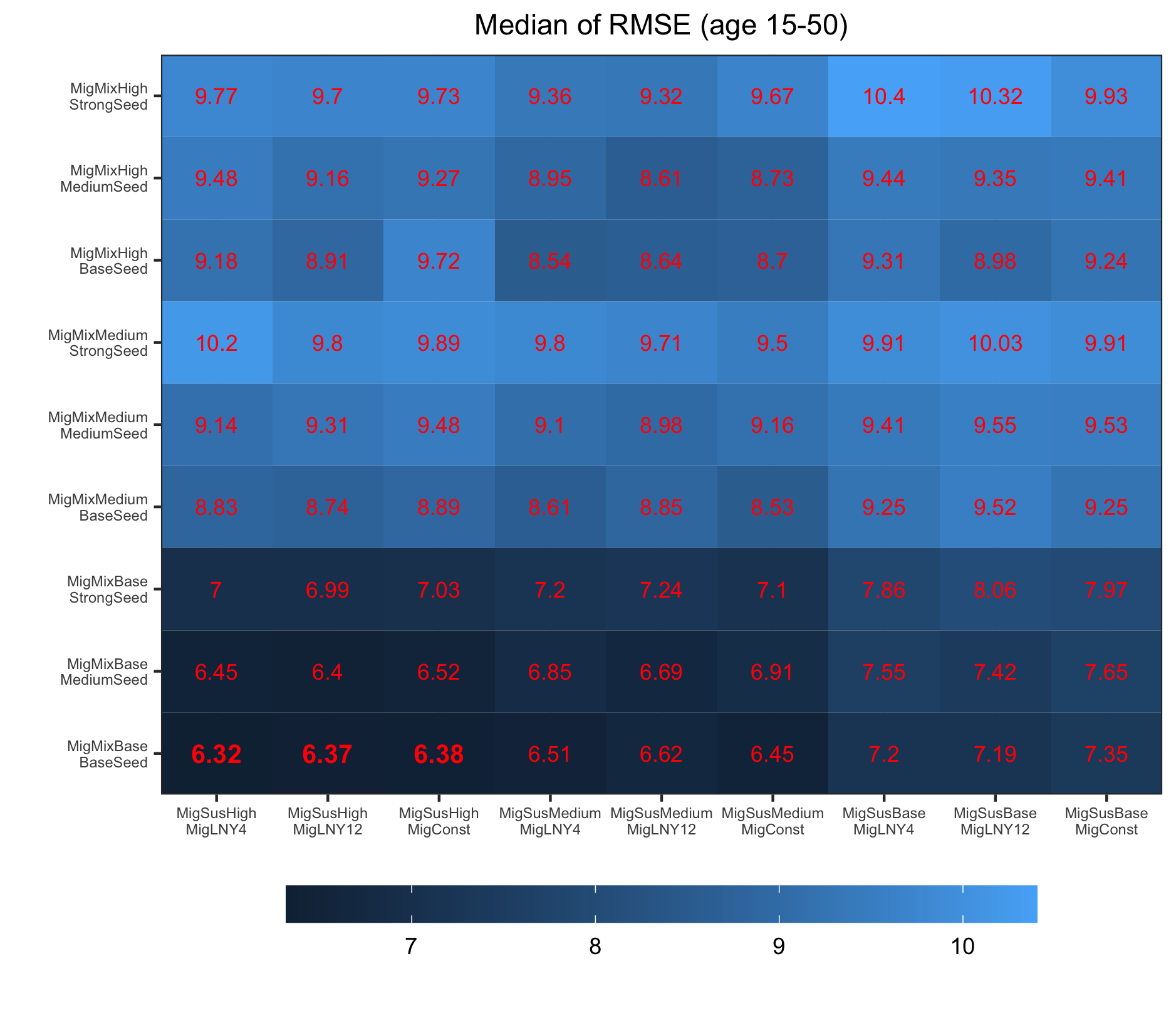


**Fig S4. Comparing migrant-related hypotheses based on medians of RMSE (age 15-50).** We formulated four sets of migrant-related hypotheses (increased migrant mixing [MigMixBase/MigMixMedium/MigMixHigh], seeding intensity [BaseSeed/MediumSeed/StrongSeed], higher migrant susceptibility [MigSusBase/MigSusMedium/MigSusHigh], and timing of migrant influx [MigConst/MigLNY12/MigLNY4]), and tested all combinations of the four sets (n=81, i.e., the number of cells in the Fig). For each model (or hypothesis combination), we conducted 100 model inference runs using a model-filter system, and show the median of the 100 RMSEs (age 15-50) in red. For infant-related hypotheses, all models tested here assumed Imm180 and InfantMixBase as baseline scenarios. See Table 1 for a summary of all hypotheses.


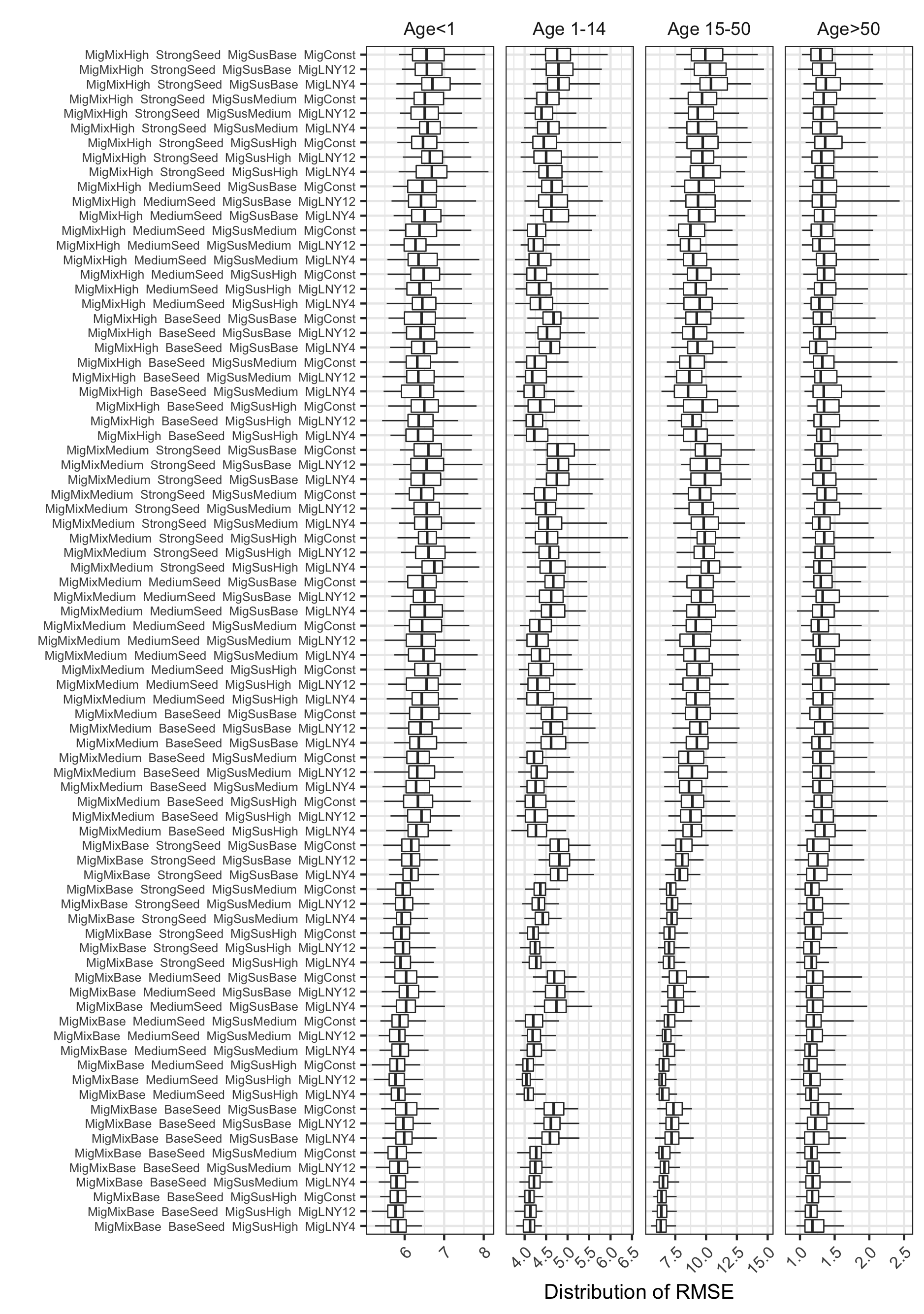


**Fig S5. Comparing migrant-related hypotheses based on distributions of RMSE.** We formulated four sets of migrant-related hypotheses (increased migrant mixing, seeding intensity, higher migrant susceptibility, and timing of migrant influx; corresponding to the 1st, 2nd, 3rd, and 4th terms in y-axis), and tested all combinations of the four sets (n=81, i.e., the number of rows of boxplots). For each model (or hypothesis combination), we conducted 100 model inference runs using a model-filter system, and show the distribution of RMSE using boxplots (whiskers = 95% interval, box edges = Interquartile range, and thick middle bar = median). For infant-related hypotheses, all models tested here assumed Imm180 and InfantMixBase as baseline scenarios. See Table 1 for a summary of all hypotheses.


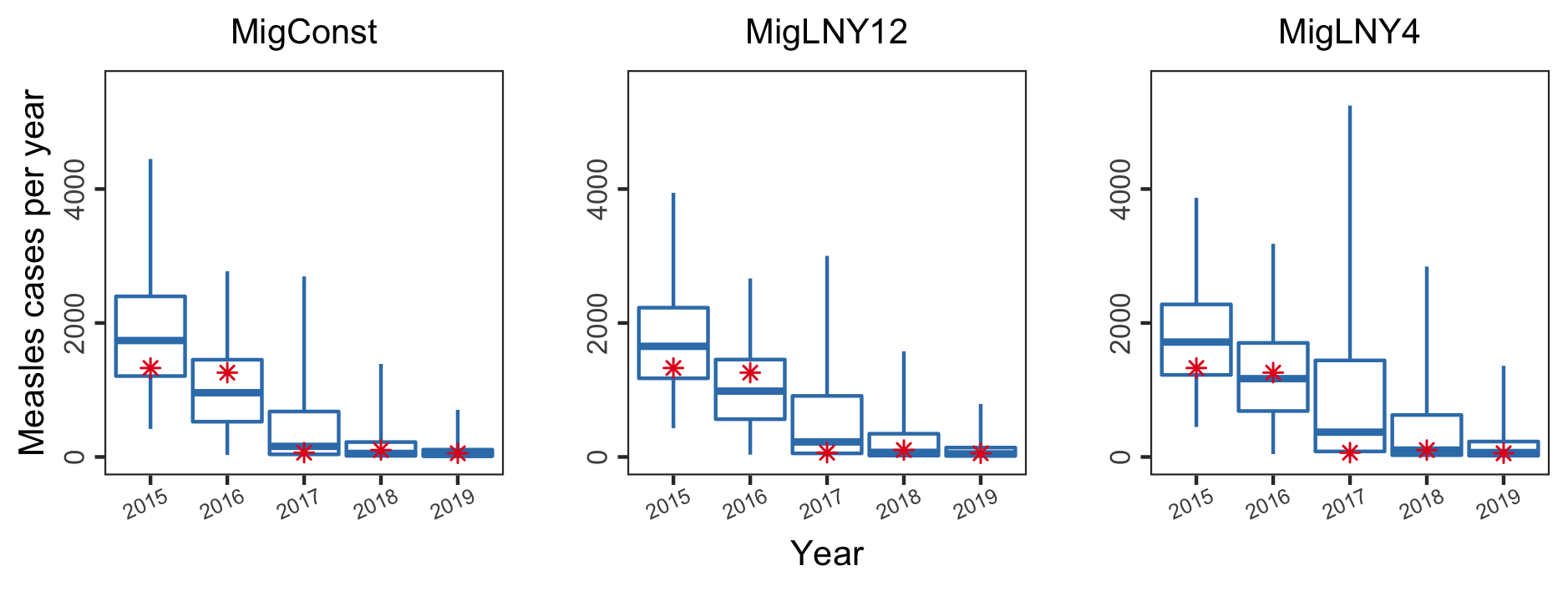


**Fig S6. Comparing migrant influx timing hypotheses based on out-of-fit prediction by year.** For the other migrant-related hypothesis settings, the three models assumed MigMixBase, BaseSeed, and MigSusHigh, because the comparison based on model fit identified this combination as the most plausible scenario. For infant-related hypotheses, the three models assumed Imm180 and InfantMixBase as baseline scenarios. See Table 1 for a summary of all hypotheses. The red asterisks represent reported measles cases for each year during 2015-2019. The boxplots represent the predicted yearly measles cases by the model replicates (8000 particles × 100 runs). The whiskers, box edges, and thick horizontal segment in the middle represent the 2.5th (or 97.5th) percentile, interquartile range, and median, respectively.


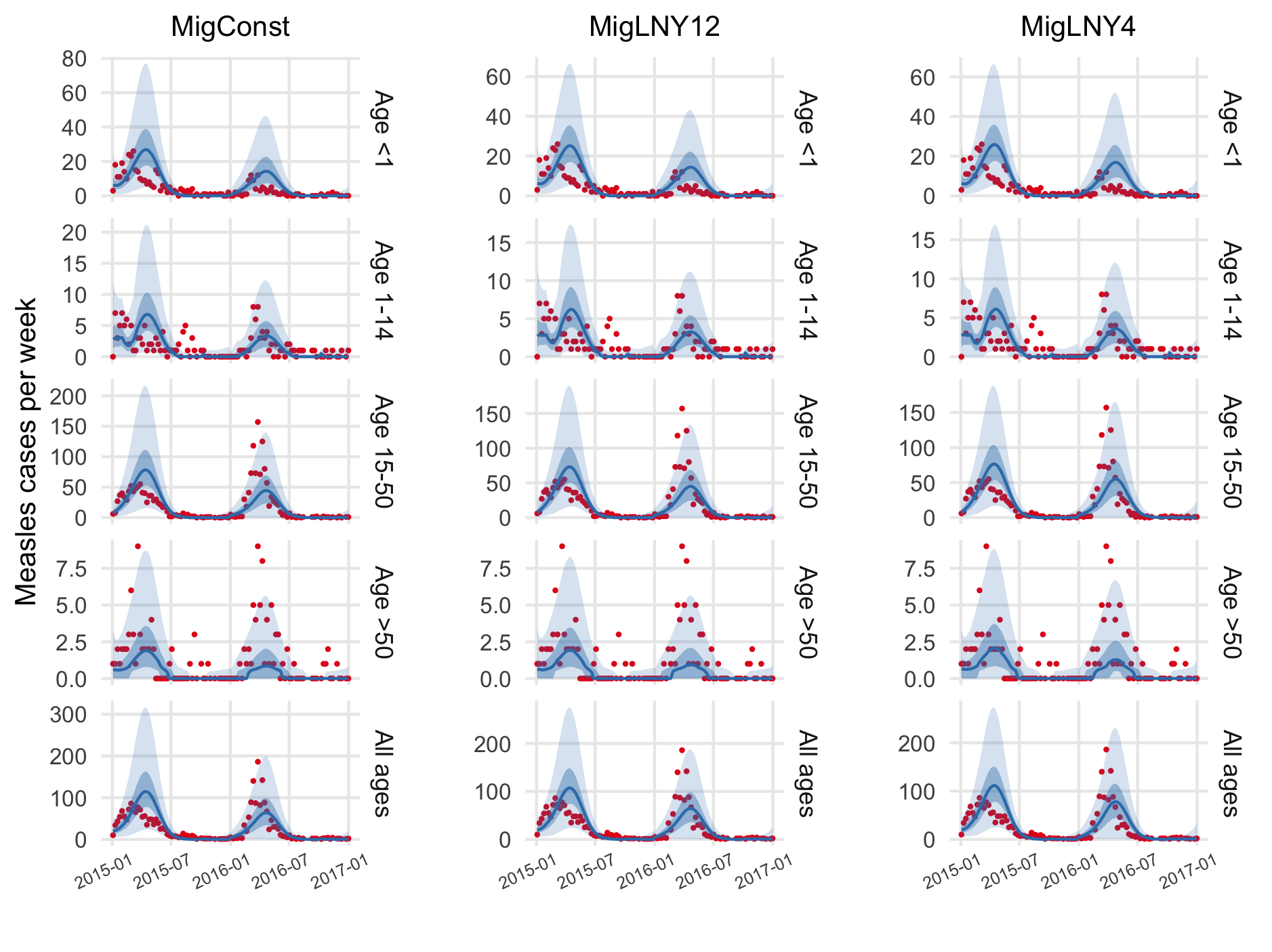


**Fig S7. Comparing migrant influx timing hypotheses based on out-of-fit prediction by age and week.** For the other migrant-related hypothesis settings, the three models assumed MigMixBase, BaseSeed, and MigSusHigh, because the comparison based on model fit identified this combination as the most plausible scenario. For infant-related hypotheses, the three models assumed Imm180 and InfantMixBase as baseline scenarios. See Table 1 for a summary of all hypotheses. The red dots represent reported measles cases for each week during 2015-2016; blue lines show the median of predicted cases; surrounding darker blue areas indicate interquartile range and lighter blue areas indicate 2.5 to 97.5 percentiles.


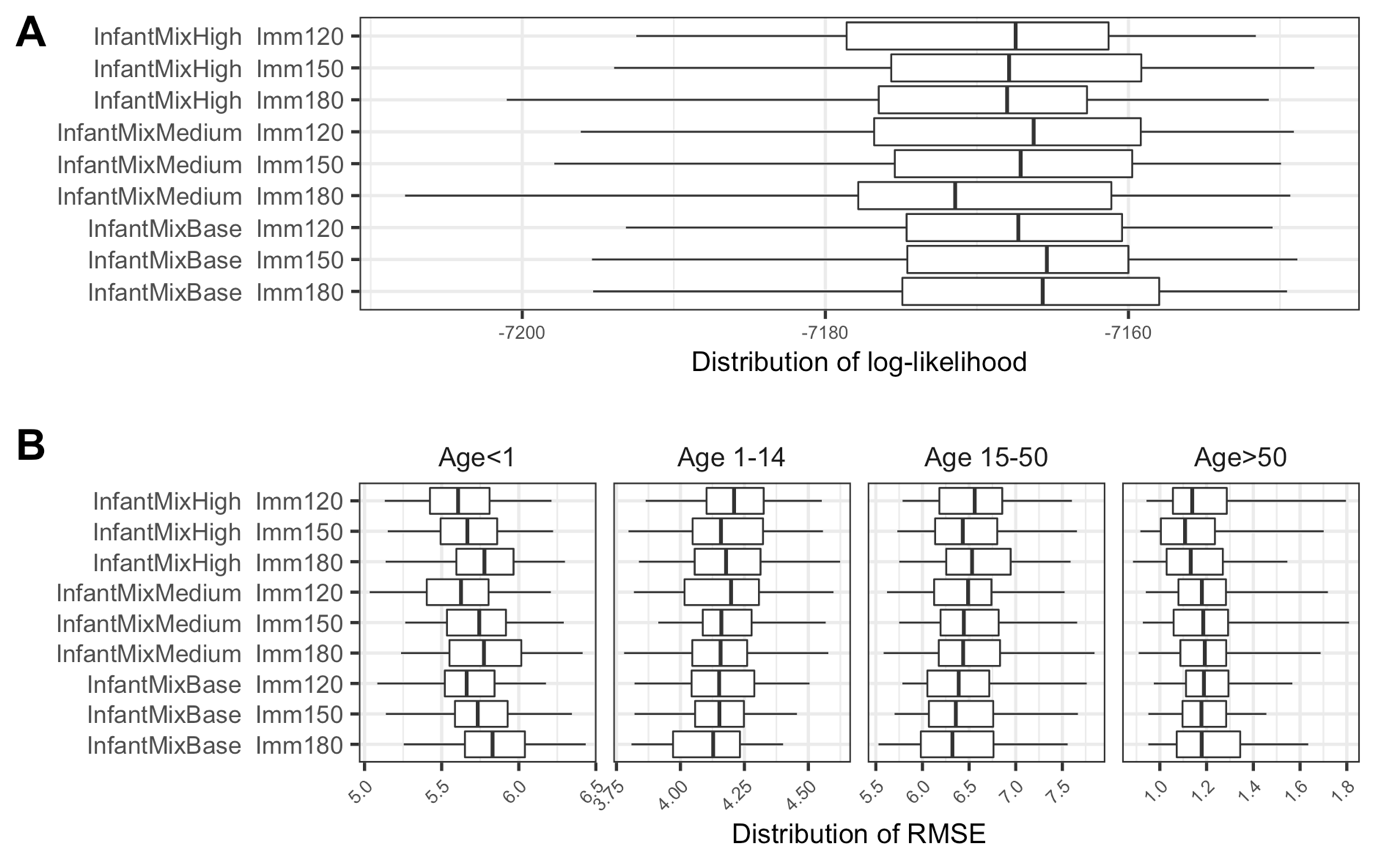


**Fig S8. Comparing infant-related hypotheses based on distributions of log-likelihood and RMSE.** We formulated two sets of infant-related hypotheses (increased infant mixing and duration of maternal immunity; corresponding to the 1st and 2nd terms in y-axis), and tested all combinations of the 2 sets (n=9, i.e., the number of rows of boxplots in each panel). For each model (or hypothesis combination), we conducted 100 model inference runs using a model-filter system, and show the distribution of log-likelihood (in **A**) and RMSE (in **B**) using boxplots (whiskers = 95% interval, box edges = Interquartile range, and thick middle bar = median). For migrant-related hypotheses, all models tested here assumed MigMixBase, BaseSeed, MigLNY4, and MigSusHigh, because the previous comparison step identified this combination as one of the most plausible scenarios. See Table 1 for a summary of all hypotheses.


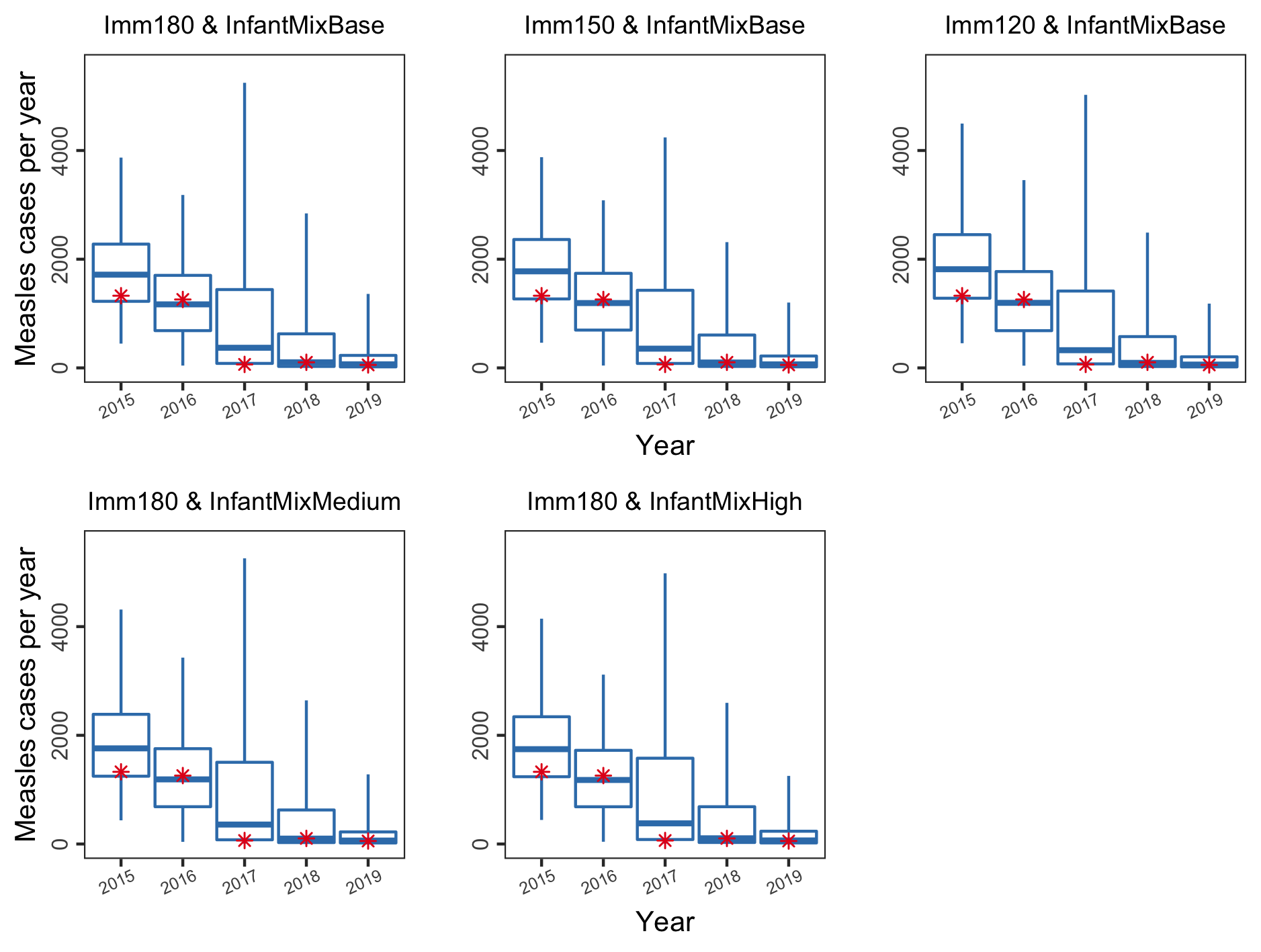


**Fig S9. Comparing infant-related hypotheses based on out-of-fit prediction by year.** For migrant-related hypotheses, all models tested here assumed MigMixBase, BaseSeed, MigLNY4, and MigSusHigh, because the previous comparison step identified this combination as one of the most plausible scenarios. See Table 1 for a summary of all hypotheses. The red asterisks represent reported measles cases for each year during 2015-2019. The boxplots represent the predicted yearly measles cases by the model replicates (8000 particles × 100 runs). The whiskers, box edges, and thick horizontal segment in the middle represent the 2.5th (or 97.5th) percentile, interquartile range, and median, respectively.


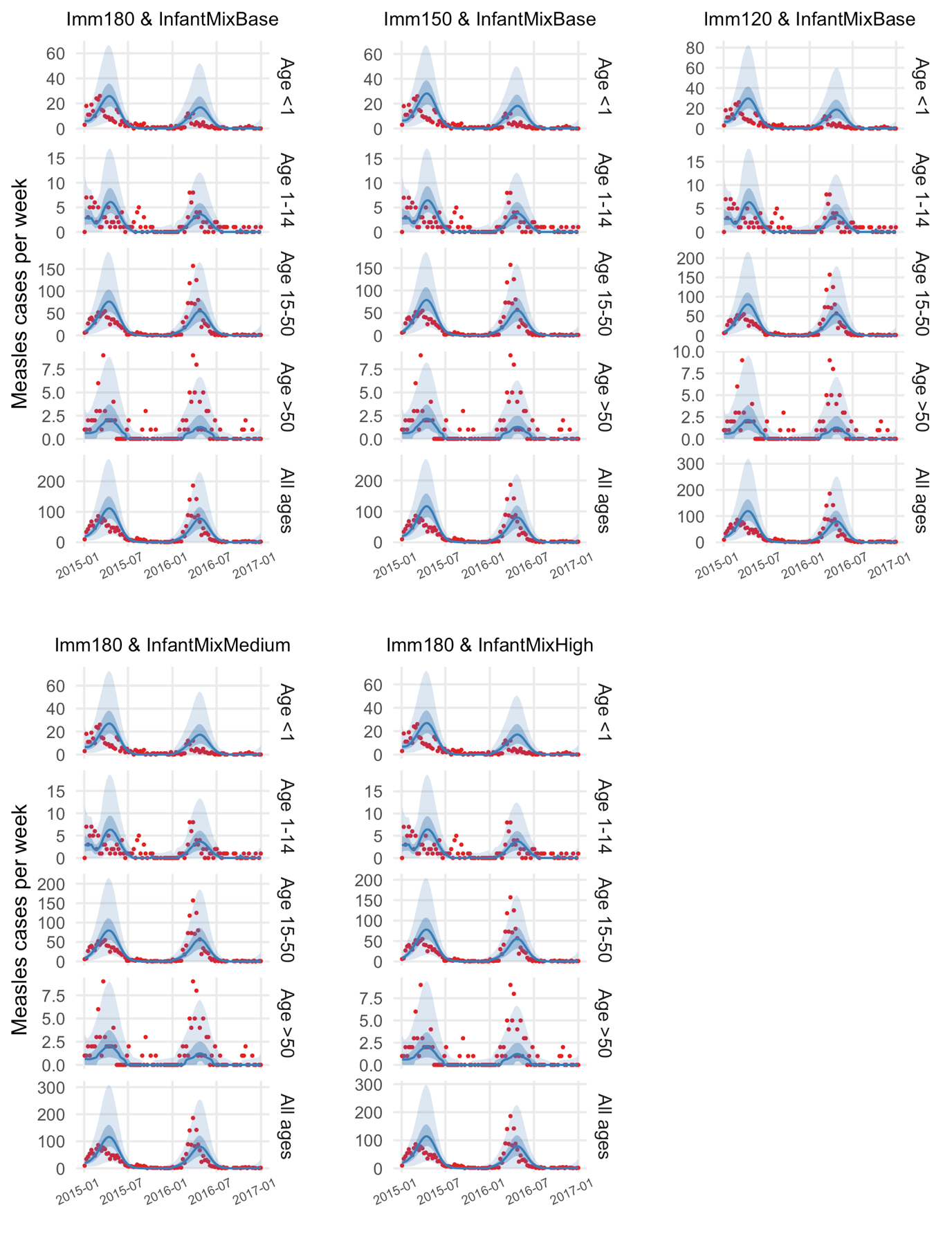


**Fig S10. Comparing infant-related hypotheses based on out-of-fit prediction by age and week.** For migrant-related hypotheses, all models tested here assumed MigMixBase, BaseSeed, MigLNY4, and MigSusHigh, because the previous comparison step identified this combination as one of the most plausible scenarios. See Table 1 for a summary of all hypotheses. The red dots represent reported measles cases for each week during 2015-2016; blue lines show the median of predicted cases; surrounding darker blue areas indicate interquartile range and lighter blue areas indicate 2.5 to 97.5 percentiles.


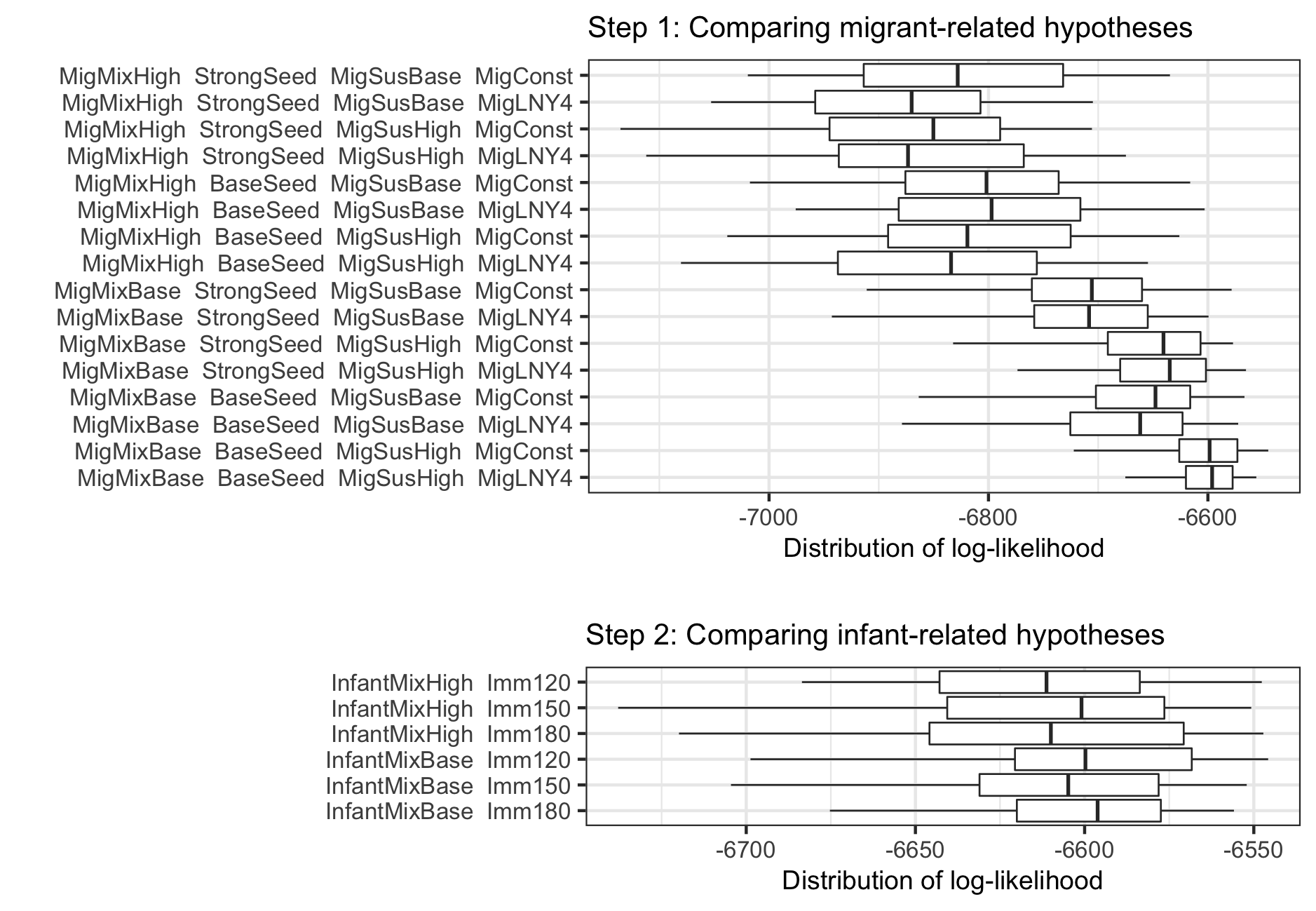


**Fig S11. Comparing hypotheses based on distributions of log-likelihood (sensitivity analysis with smaller observational error).** The upper and lower panels were the same with Figs S3 and S8A, respectively, except that we used smaller observational errors in model inference and tested fewer combinations of hypotheses.


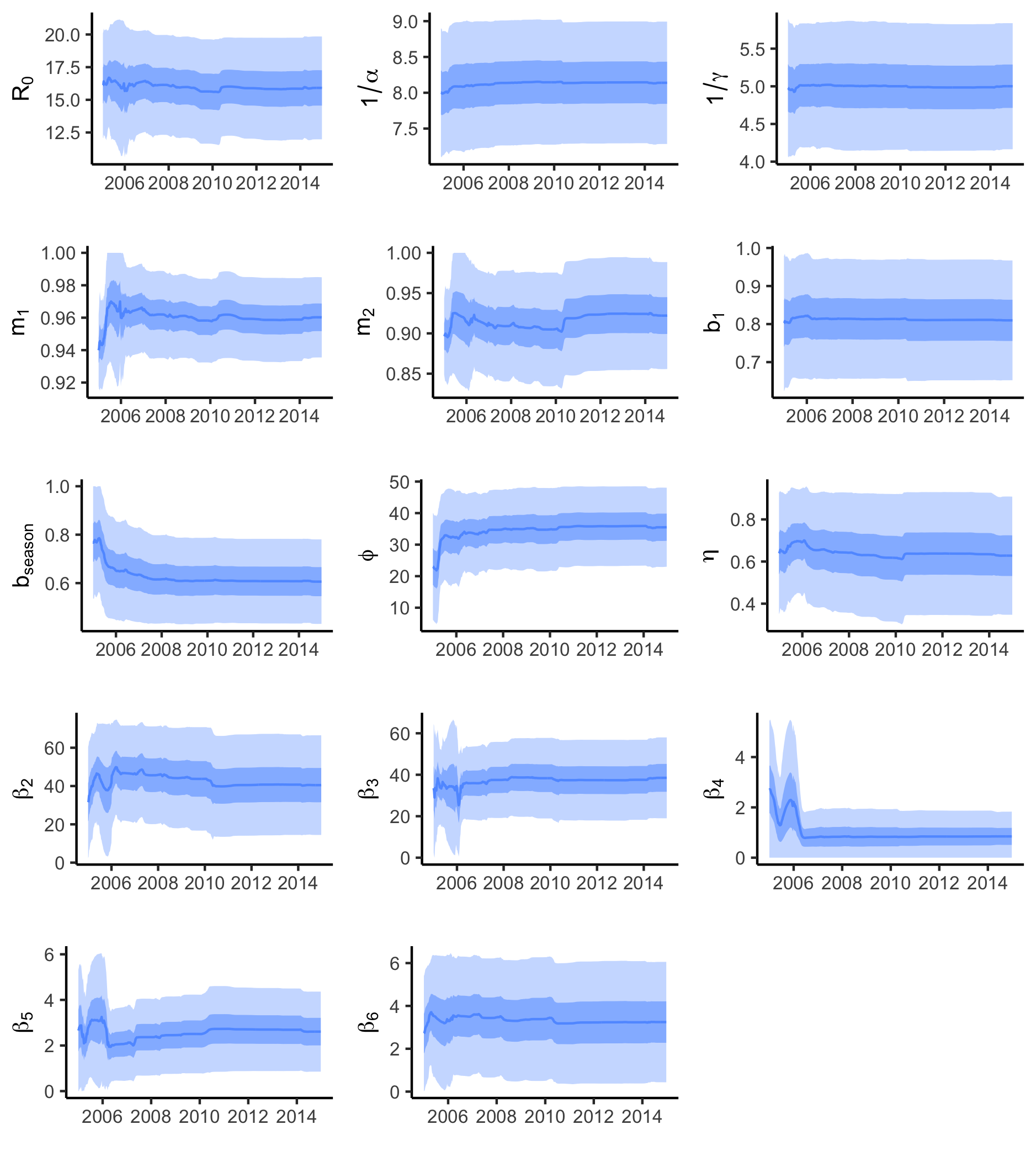


**Fig S12. Estimates of model parameters.** The model assumed MigSusHigh, BaseSeed, MigLNY4, MigMixBase, Imm180, and InfantMixBase (see Table 1 for a summary of all hypotheses). Blue lines and surrounding regions show the means and 50% and 95% credible intervals of estimates. $R_{0}$ represents basic reproductive number; $1/\alpha$, duration of the exposed state; $1/\gamma$, duration of the infectious state; $m_{1}$ and $m_{2}$, mixing parameters; $b_{1}$ and $b_{season}$, amplitudes of school term forcing and sinusoidal seasonal forcing, respectively; $\phi$, the day of a year when the sinusoidal forcing reaches the maximum; $\eta$, reporting rate in measles surveillance; $\beta_{i}$ ($i\in\{2,3,4,5,6\}$), relative transmission rates.


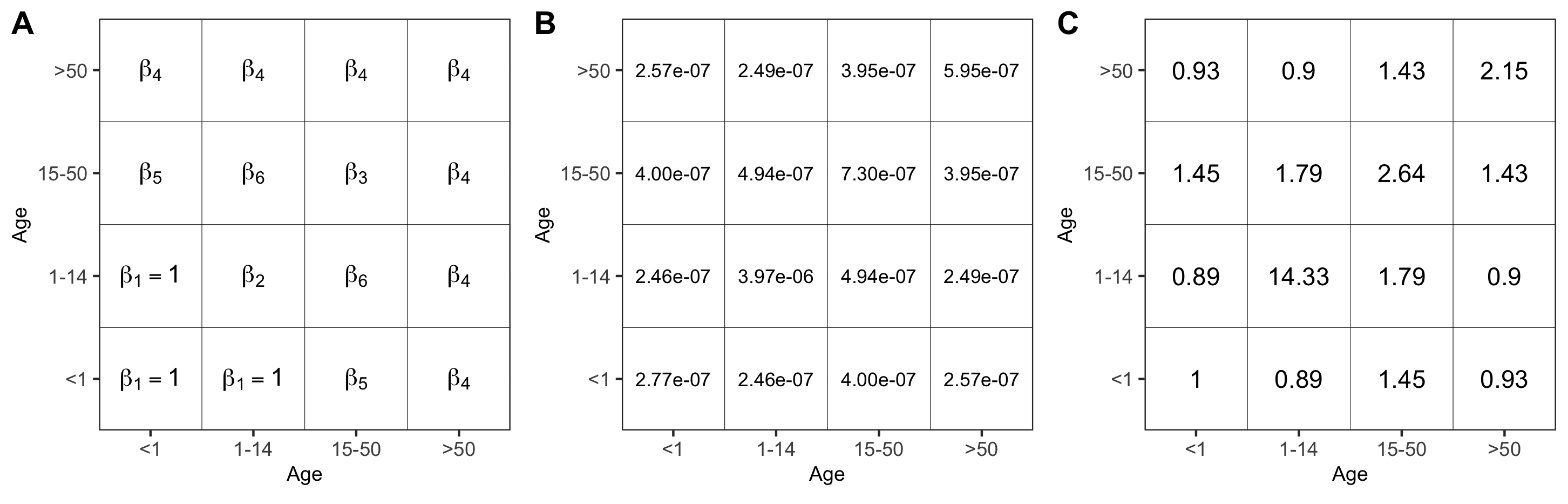


**Fig S13. Using contact data from the literature to inform the priors of** $\boldsymbol{\beta}_{\boldsymbol{2}}$ **to** $\boldsymbol{\beta}_{\boldsymbol{6}}$**.** In our model, $\beta$ parameters (see **A**) govern mixing among age groups. Because we estimated the magnitude of $\beta_{2}$ to $\beta_{6}$ relative to $\beta_{1}$, we divided all $\beta$ parameters by $\beta_{1}$, thus setting $\beta_{1}$ to 1. Based on the contact data generated by Mistry et al., we derived a contact matrix (see **B**), with each element representing the contact rate between two age groups. To match the Mistry’s matrix (**B**) with our matrix (**A**), we divided the Mistry’s matrix by the element on Row 1 and Column 1, and used these scaled elements (see **C**) to inform the priors used.
