# Supplementary material for: Determinants of measles persistence in Beijing, China: A modelling study
Source: Epidemiol Infect. 2023 Aug 22;151:e144. doi: 10.1017/S0950268823001322 (PMC10540187; doi:10.1017/S0950268823001322)
Supplement: Chen et al. supplementary material [file S0950268823001322sup002.docx]

Supplementary Materials - Determinants of measles persistence in Beijing, China: A modeling study

Table of Contents

[1 Demographic data and estimates 2](#_Toc128398371)

[1.1 Birth and death 2](#_Toc128398372)

[1.2 Migration 2](#_Toc128398373)

[2 Vaccination data and estimates 2](#_Toc128398374)

[2.1 Routine vaccination program 2](#_Toc128398375)

[2.2 Catch-up or supplementary vaccination programs 2](#_Toc128398376)

[2.2.1 Catch-up vaccination program for pre-school children of migrant workers 3](#_Toc128398377)

[2.2.2 Catch-up vaccination program for migrant workers 3](#_Toc128398378)

[2.2.3 The 2010 synchronized nationwide supplementary immunization activity (SIA) 3](#_Toc128398379)

[3 Priors of model state 3](#_Toc128398380)

[4 Hypotheses tested and model settings 4](#_Toc128398381)

[4.1 Higher migrant susceptibility 4](#_Toc128398382)

[4.2 Case importation due to migrant influx 5](#_Toc128398383)

[4.3 Timing of migrant influx 5](#_Toc128398384)

[4.4 Increased migrant mixing 5](#_Toc128398385)

[4.5 Shorter duration of maternal immunity 6](#_Toc128398386)

[4.6 Increased infant mixing 6](#_Toc128398387)

[References 6](#_Toc128398388)

# 1 Demographic data and estimates

## 1.1 Birth and death

Yearly data from 1995 to 2019 on birth and death rates were compiled from the Beijing Statistical Yearbook [1]. Yearly infant death rates during this period were available from the Beijing Municipal Commission of Health and Family Planning [2]. We calculated the death rates for those aged 1-14, 15-50, and >50 based on the age-specific urban death rates from the national census in 2010 [3].

## 1.2 Migration

We calculated the yearly net numbers of migrants in Beijing according to

$$\begin{aligned} migration_{y}=population_{y}-population_{y-1}-population_{y}\times\left( birth.rate_{y}-death.rate_{y} \right)\#\left( 1 \right) \end{aligned}$$

where $y$ is the calendar year. The term $population_{y}$ represents the total population in Beijing in Year $y$ [1]. Because the total population increased in most years during the study period, estimated net migration was positive in most of the years. Based on the age structure of migrants in 2010 [4], we calculated the yearly net migration in those aged <1, 1-14, 15-50, >50 years, i.e., the four age groups in our model. Using the yearly migration, we further calculated daily migration, and the calculation varied by the tested hypothesis (Section 4.3, Supplementary Materials).

# 2 Vaccination data and estimates

## 2.1 Routine vaccination program

In Equation (1) of the main text, $v^{r}(t)$ represents the proportion of susceptibles acquiring immunity from routine vaccination; for simplicity, we included only 1 vaccine dose at 1 year old to represent the combined outcome of the first and the second dose at 8 months and 18-24 months of age [5]. To include the vaccination coverage and vaccine efficacy for both doses, we calculated $v^{r}(t)$ as

$$\begin{aligned} v^{r}\left( t \right)=VC_{2}\times VE_{2}+\left( VC_{1}-VC_{2} \right)\times VE_{1}\#\left( 2 \right) \end{aligned}$$

where $VC_{i}$ and $VE_{i}$ represent the vaccination coverage and efficacy for 1 and 2 doses. For the period of 1995-2011, when vaccination data were available, the coverage of 1 and 2 doses was generally above 99% [5]; thus, for years after 2011, we assumed the coverage was the same as that in 2011. We set $VE_{1}$ and $VE_{2}$ to 85% and 95% based on the estimates of the efficacy of the measles vaccine in China [6,7], which were consistent with the WHO position paper on measles vaccines (84% for $VE_{1}$ and 95% for $VE_{2}$) [8].

## 2.2 Catch-up or supplementary vaccination programs

In Equation (1) of the main text, $v_{i}^{c}(t)$ represents the rate (persons per day) at which the various catch-up or supplementary vaccination programs confer immunity to population included in the catch-up vaccination programs.

### 2.2.1 Catch-up vaccination program for pre-school children of migrant workers

From 2000 onwards, a catch-up vaccination program was conducted for the pre-school children (<7 years; Group 2) of migrant workers [9]. The program was conducted from March to May each year, and targeted those without a measles vaccination record. The numbers of vaccinees during 2005-2010 were obtained from the literature [9]. We calculated $v_{2}^{c}(t)$ for the program period by assuming that (i) the vaccinees were equally distributed across the days and (ii) the single dose was 90% effective. Of note, for years when vaccination data were unavailable (i.e., 2000-2004 & from 2011 onwards), we assumed that the number of vaccinees scaled with the number of migrants coming to Beijing.

### 2.2.2 Catch-up vaccination program for migrant workers

From 2005 onwards, a catch-up vaccination program was conducted for migrant workers (most of them aged 15-50; Group 3) [9]. The program was also conducted from March to May and migrants were vaccinated regardless of their vaccination history. The numbers of vaccinees during 2005-2010 were also obtained from the literature [9]. We calculated $v_{3}^{c}(t)$ for the program period by assuming that (i) vaccinees were equally distributed across the days, (ii) only those susceptible prior to the catch-up vaccination were immunized, (iii) the vaccinees had the same susceptibility levels as the migrants coming to Beijing, and (iv) the single dose was 90% effective. For years (from 2011 onwards) when catch-up vaccination data were unavailable, we assumed that the number of vaccinees scaled with the number of migrants coming to Beijing.

### 2.2.3 The 2010 synchronized nationwide supplementary immunization activity (SIA)

To support the goal of measles elimination by 2012, from September 11th to 20th in 2010, a synchronized SIA for measles was conducted nationwide, including in Beijing [9,10]. This SIA targeted the general population aged 8 months to 14 years regardless of vaccination history. In Beijing, 1624998 children received one dose of vaccine [9]. Of the vaccinees, we assumed that $\frac{(1-\frac{2}{3})}{(14-\frac{2}{3})}$ and $\frac{(14-1)}{(14-\frac{2}{3})}$ were in the age range of 8-12 months (part of Group 1) and 1-14 years (Group 2), respectively. We then calculated $v_{1}^{c}(t)$ and $v_{2}^{c}(t)$ by assuming that (i) the 1624998 doses were equally distributed across those 10 days, (ii) only those susceptible prior to the SIA were immunized, (iii) vaccinee susceptibility levels equaled the model-estimated susceptibility levels for the age groups, and (iv) the single dose was 90% effective.

# 3 Priors of model state

For each inference run, we initialized the model state (referring to the state variables and parameters) at the start of 1995 using pre-specified priors. We used the estimated posterior distribution at the end of 1994 in our previous study [11] to set the priors of the model state in the current analysis, except for $\phi$ and $\beta_{2}$ to $\beta_{6}$.

$\phi$ represents the day of a year when the sinusoidal forcing reached the maximum amplitude, and was fixed at 23 days in our previous study [11]. Here, we set the prior of $\phi$ to be a uniform distribution between 3 and 43 days, considering that measles outbreaks typically start in early spring.

For $\beta_{2}$ to $\beta_{6}$, which govern the mixing among age groups, we set the priors based on a contact matrix provided by Mistry et al. for Beijing [12] (see Fig S13 for details). We show the specific priors used in Table S1. Here, we allowed for wide prior ranges, particularly for $\beta_{2}$ and $\beta_{3}$ to account for strong mixing among school children and among migrant workers.

# 4 Hypotheses tested and model settings

## 4.1 Higher migrant susceptibility

We formulated three hypotheses related to the population susceptibility among migrants coming to Beijing (MigSusBase, MigSusMedium, and MigSusHigh). These hypotheses correspond to different specifications of $f_{i}^{S}(t)$, $f_{i}^{E}(t)$, $f_{i}^{I}(t)$, and $f_{i}^{R}(t)$ in Equation (1) of the main text, which represent the proportions of susceptible, exposed, infectious, and recovered individuals among migrants in the $i$th age group at time $t$, respectively.

Hypothesis MigSusBase assumed migrants were as susceptible to measles as the population in Beijing; specifically, $f_{i}^{[.]}(t)$ was set to $\frac{[.]_{i}}{N_{i}}$ where $[.]\in\{S,E,I,R\}$ and $\frac{[.]_{i}}{N_{i}}$ is the proportion of susceptible, exposed, infectious, or recovered individuals in the population of Beijing. Hypotheses MigSusMedium and MigSusHigh assumed migrants were as susceptible as and 50% more susceptible than the population of Shandong, respectively; specifically,

$$\begin{aligned} f_{i}^{S}\left( t \right)=\left\{ \begin{matrix} \frac{S_{i}^{SD}}{N_{i}^{SD}} & \mathrm{if}i\in\left\{ 1,4 \right\} \\ \rho\frac{S_{i}^{SD}}{N_{i}^{SD}} & \mathrm{if}i\in\left\{ 2,3 \right\} \end{matrix} \right., f_{i}^{E}\left( t \right)=\frac{E_{i}^{SD}}{N_{i}^{SD}}, f_{i}^{I}\left( t \right)=\frac{I_{i}^{SD}}{N_{i}^{SD}}\#\left( 5 \right) \end{aligned}$$

$\frac{[.]_{i}^{SD}}{N_{i}^{SD}}$ ($[.]\in\{S,E,I\}$) is the proportion of susceptible, exposed, or infectious individuals in the population of Shandong. For MigSusMedium and MigSusHigh, $\rho$=1 and 1.5, respectively. For all hypotheses, $f_{i}^{R}(t)=1-f_{i}^{S}(t)-f_{i}^{E}(t)-f_{i}^{I}(t)$.

We note two further points below. First, we applied the adjustment $\rho$ only to Groups 2 and 3 because the susceptibility difference was likely most pronounced among these two age groups; recent routine EPI programs (for Group 1) were of high coverage in most regions and most of those above 50 years (Group 4) had natural infections. Second, we used estimates of population susceptibility of Shandong from our previous research as a proxy for migrant susceptibility [11]. These estimates were available for a part of our study period, i.e., 1995-2004. For 2005-2019, we assumed the migrant population susceptibility was the same with that in 2004. However, because a nationwide SIA was conducted in 2010, during which about half of children aged 8 months to 14 years across China were vaccinated regardless of vaccination history, we assumed the migrant population susceptibility for those aged 1-14 decreased by half after that SIA.

## 4.2 Case importation due to migrant influx

We formulated three hypotheses related to case importation (seeding) via migration (BaseSeed, MediumSeed, and StrongSeed). These hypothese correspond to different specifications of $h_{i}(t)$ in Equation (1) of the main text; $h_{i}(t)$ represents the number of imported cases for the $i$th age group at time $t$.

For BaseSeed, $h_{i}(t)$ was set to 1 for $i\in\left\{ 1,2,3 \right\}$ on the 8th day after the LNY and on October 1st (the national day). Both days are major holidays in China with substantial travel. For MediumSeed and StrongSeed, we considered case importation on top of the BaseSeed scenario on the 8th to 10th day after the LNY. For those days, we added $MigPopScalingFactorp_{i}$ to $h_{i}(t)$. $MigPop$ represents yearly net migrant influx; $ScalingFactor$=${10}^{-5}$ and $2{10}^{-5}$ for MediumSeed and StrongSeed, respectively; $p_{i}$=0, 1/3, 2/3, and 0 for Groups 1-4 (those aged <1, 1-14, 15-50, and >50), respectively. For MediumSeed, the mean of daily imported case counts was 3.6 (averaging across the years for all ages combined); for StrongSeed, the corresponding mean was 7.3.

## 4.3 Timing of migrant influx

We formulated three hypotheses related to the timing of migrant influx to Beijing (MigConst, MigLNY4, and MigLNY12). These hypotheses correspond to different specifications of $r_{i}(t)$ in Equation (1) of the main text; $r_{i}(t)$ represents the net migration for the $i$th age group at time $t$.

For MigConst, $r_{i}(t)$ was set to 1/365 times the yearly net number of migrants in Group $i$ in that year, i.e., constant migration. For MigLNY4, the total number of net migrants for each year was distributed mostly during Weeks 2-4 following the Lunar New Year (LNY) per a gamma distribution (mean = 7 days and SD = 3 days; assuming migration started in Week 2). Using this distribution, the influx peaked at the end of Week 2 and decreased to about 0 at the end of Week 4. The MigLNY12 scenario was similar: Using another gamma distribution (mean = 28 days and SD = 20 days), and the influx started in Week 2, peaked at the end of Week 3, and decreased to about 0 at the end of Week 12.

## 4.4 Increased migrant mixing

We formulated three hypotheses related to increased mixing intensity among migrants during times with high migrant influx (MigMixBase, MigMixMedium, and MigMixHigh). These hypotheses correspond to different specifications of $k_{3}(t)$ in Equation (3) of the main text. $k_{i}(t)$ allows the mixing intensity to vary by age groups $i$ or over time $t$. For this analysis, we set $k_{i}(t)=1$ for $i\in\{2,4\}$ and for all $t$ in all tested hypotheses. The specification of $k_{1}(t)$ is detailed in Section 4.6 (increased infant mixing) below.

For MigMixBase, we set $k_{3}(t)=1$ for all $t$, meaning no change over time in mixing intensity among Group 3 (15-50 years; the age group for most migrants). For MigMixMedium and MigMixHigh,

$$\begin{aligned} k_{3}\left( t \right)=\left\{ \begin{matrix} k_{0} & \mathrm{if}t\in T_{shortly after LNY} \\ 1 & \mathrm{if}t\notin T_{shortly after LNY} \end{matrix} \right..\#\left( 6 \right) \end{aligned}$$

For MigMixMedium and MigMixHigh, $k_{0}$=1.025 and 1.05, respectively. $T_{shortly after LNY}$ was 7 days after the LNY to March 31st.

Of note, because the prior of $m_{1}$ and $m_{2}$ (in Equation (3) of the main text) ranged from about 0.90 to 0.95, scaling these values by 1.05 (i.e., $k_{0}$=1.05) would lead the mixing parameter to approximate 1, which represents perfect mixing. Therefore, we did not test larger values of $k_{0}$. The same rationale applies to the setting of $k_{1}(t)$ in the increased infant mixing hypotheses.

## 4.5 Shorter duration of maternal immunity

We formulated three hypotheses with different mean durations of maternal immunity (Imm180, Imm150, and Imm120). These hypotheses correspond to different specifications of $p(t)$ in Equation (1) of the main text. Here $p(t)$ is given by a logistic function: $180-\frac{180-p_{0}}{1+e^{-0.5(y-2006)}}$. With this formulation, the mean duration of maternal immunity decreases gradually over time from 180 days to $p_{0}$ with the midpoint being the year of 2006. For Imm180, Imm150, and Imm120, $p_{0}$ was set to 180, 150, and 120 days, respectively. We set the midpoint to 2006 because the formal establishment of the routine vaccination program was in 1978 [5], and a woman born in that year reached a typical child-bearing age (i.e., 28 years old) in 2006.

## 4.6 Increased infant mixing

We formulated three hypotheses related to the mixing intensity among those <1 year: (i) $k_{1}(t)$ in Equation (3) of the main text was set to 1.0 (InfantMixBase); (ii) $k_{1}(t)$ set to 1.025 (InfantMixMedium); and (iii) $k_{1}(t)$ set to 1.05 (InfantMixHigh).

# References

1. Beijing statistical yearbook 2020. Available: <http://nj.tjj.beijing.gov.cn/nj/main/2020-tjnj/zk/indexeh.htm>

2. Infant mortality rate in beijing from 1949 to 2020. Available: <http://www.phic.org.cn/tjsj/wssjzy/jkzb/202104/t20210409_295833.html>

3. The sixth census of the people’s republic of china. Available: <http://www.stats.gov.cn/tjsj/pcsj/rkpc/5rp/index.htm>

4. Tabluation of the 2010 population census of beijing municipality. Available: <http://nj.tjj.beijing.gov.cn/tjnj/rkpc-2010/indexch.htm>

5. Li J, Lu L, Pang X, Sun M, Ma R, Liu D, et al. A 60-year review on the changing epidemiology of measles in capital beijing, china, 1951-2011. BMC Public Health. 2013;13: 986. doi:[10.1186/1471-2458-13-986](https://doi.org/10.1186/1471-2458-13-986)

6. Dai B, Chen Z, Liu Q, Wu T, Guo C, Wang X, et al. Duration of immunity following immunization with live measles vaccine: 15 years of observation in zhejiang province, china. Bulletin of the World Health Organization. 1991;69: 415.

7. Yang Z, Xu J, Wang M, Di B, Tan H, He Q, et al. Measles epidemic from 1951 to 2012 and vaccine effectiveness in guangzhou, southern china. Hum Vaccin Immunother. 2014;10: 1091–6. Available: <http://www.ncbi.nlm.nih.gov/pubmed/24513504>

8. Measles vaccines: WHO position paper–April 2017. Available: https://www.who.int/publications-detail-redirect/WER9217.

9. Li J, Lu L, Liu D, Ma R, Wu J, Pang X. Measles epidemiology and elimination measure evaluation in beijing, 2005-2010. Disease Surveillance. 2012; 353–357. doi:[10.3784/j.issn.1003-9961.2012.5.007](https://doi.org/10.3784/j.issn.1003-9961.2012.5.007)

10. Ma C, Rodewald L, Hao L, Su Q, Zhang Y, Wen N, et al. Progress toward measles elimination - china, january 2013-june 2019. MMWR Morb Mortal Wkly Rep. 2019;68: 1112–1116. doi:[10.15585/mmwr.mm6848a2](https://doi.org/10.15585/mmwr.mm6848a2)

11. Yang W, Li J, Shaman J. Characteristics of measles epidemics in China (1951-2004) and implications for elimination: A case study of three key locations. Plos Comput Biol. 2019;15(2):e1006806.

12. Mistry D, Litvinova M, Pastore y Piontti A, et al. Inferring highresolution human mixing patterns for disease modeling. Nat Commun. 2021;12(1):323. doi:10.1038/s41467-020-20544-y
