# Supplementary material for: Determinants of measles persistence in Beijing, China: A modelling study
Source: Epidemiol Infect. 2023 Aug 22;151:e144. doi: 10.1017/S0950268823001322 (PMC10540187; doi:10.1017/S0950268823001322)
Supplement: Chen et al. supplementary material [file S0950268823001322sup003.docx]

| **Table S1. The prior used for estimating** $\boldsymbol{\beta}_{\boldsymbol{2}}$ **to** $\boldsymbol{\beta}_{\boldsymbol{6}}$**.** | | | |
| --- | --- | --- | --- |
|  | Interpretation (the level of contact between age groups) | Values according to the contact matrix by Mistry et al. See Fig S12 for details. | Prior used |
| $\beta_{1}$ | Among <1-year-olds | 1 or 0.89 | Fixed to 1^a^ |
| $\beta_{2}$ | Among 1- to 14-year-olds | 14.33 | U [5, 60] |
| $\beta_{3}$ | Among 15- to 50-year-olds | 2.64 | U [0.5, 60] |
| $\beta_{4}$ | Between >50-year-olds and all age groups | 0.93, 0.90, 1.43, or 2.15 | U [0.1, 5] |
| $\beta_{5}$ | Between <1-year-olds and 15- to 50-year-olds | 1.45 | U [0.1, 5] |
| $\beta_{6}$ | Between 1- to 14-year-olds and 15- to 50-year-olds | 1.79 | U [0.1, 5] |
| ^a^ Because we estimated the magnitude of $\beta_{2}$ to $\beta_{6}$ relative to $\beta_{1}$, we fixed the value of $\beta_{1}$ to 1 in the model. | | | |
